# From scaffold to function: A systematic review on dLECM-based hydrogels for liver tissue engineering — Fabrication, properties, and translational applications

**DOI:** 10.1016/j.reth.2025.11.006

**Published:** 2025-11-18

**Authors:** Ziba Majidi, Mohammad Mahdi Sarvi, Mohammad Aref Rajabi, Houran Firouzian, Iman Seyhoun, Masoumeh Majidi Zolbin

**Affiliations:** a Department of Medical Laboratory Science, School of Allied Medical Sciences, Tehran University of Medical Sciences, Tehran, Iran; b School of Medicine, Tehran University of Medical Sciences, Tehran, Iran; c Autonomous Nervous System (ANS) Association, Students' Scientific Research Center (SSRC), Tehran University of Medical Sciences, Tehran, Iran; d Iranian Tissue Bank and Research Center, Tehran University of Medical Sciences, Tehran, Iran; e Department of Applied Cell Sciences, School of Advanced Technologies in Medicine, Tehran University of Medical Sciences, Tehran, Iran

**Keywords:** Decellularization, Extracellular matrix, Hydrogel, Liver tissue engineering, Systematic review, Biomaterials

## Abstract

**Background:**

Liver tissue engineering is a rapidly advancing field aiming to address the critical shortage of donor organs and improve *in vitro* models for drug screening and disease modeling. Decellularized liver extracellular matrix (dLECM)-based hydrogels have emerged as a leading biomaterial platform due to their ability to preserve the native biochemical composition, microstructure, and biomechanical cues of the liver microenvironment. This systematic review aims to comprehensively evaluate the methodologies, physicochemical properties, biological performance, and translational applications of dLECM-derived hydrogels in liver tissue engineering, with a focus on fabrication protocols, functional outcomes, and challenges toward clinical implementation.

**Methods:**

A systematic search was conducted in accordance with PRISMA 2020 guidelines across PubMed, Scopus, Google scholar and Web of Science. A total of 74 studies were included after screening 536 records identified from databases. Data were extracted on tissue source, decellularization techniques, dECM solubilization, hydrogel formulation, crosslinking methods, physicochemical characterization, and *in vitro*/*in vivo* functional outcomes.

**Results:**

The majority of studies utilized porcine or rodent livers, with immersion/agitation and vascular perfusion as the primary decellularization methods. Sodium dodecyl sulfate (SDS), Triton X-100, and ammonium hydroxide were the most common detergents, often combined with enzymatic treatments (e.g., DNase, trypsin) to enhance nuclear removal. dLECM was predominantly solubilized using pepsin in acidic conditions (acetic acid or HCl) and reconstituted into hydrogels via thermal gelation at 37 °C. The resulting hydrogels demonstrated excellent biocompatibility, supporting high viability and enhanced functional activity—including albumin secretion, urea synthesis, and expression of hepatic markers (e.g., CYP450, CK18, AFP)—in primary hepatocytes, HepG2 cells, and stem cell-derived hepatocyte-like cells. Advanced applications such as 3D bioprinting, organoid culture, and *in vivo* transplantation in liver injury models (e.g., CCl_4_-induced fibrosis, acute liver failure) further highlight the therapeutic potential of these biomaterials. However, significant heterogeneity was observed in decellularization efficacy, residual DNA content (ranging from 1.04 % to 21.74 ng/mg), and mechanical characterization, with many studies lacking standardized reporting of storage modulus (G′) or gelation kinetics.

**Conclusion:**

dLECM-based hydrogels represent a highly promising and biomimetic platform for liver tissue engineering, capable of supporting complex cellular functions and regenerative outcomes. Despite significant progress, standardization of fabrication protocols, comprehensive physicochemical characterization, and long-term *in vivo* safety assessments are essential to advance these materials toward clinical translation.

## 1 Introduction

The liver isn't just the largest internal organ in the human body—it's also one of the most resilient. Packed with essential functions like detoxifying harmful substances, synthesizing proteins, producing bile, and managing metabolism, it can bounce back from injuries ranging from drug toxicity to partial surgical removal. This natural regenerative ability, driven largely by hepatocytes and their intricate crosstalk with other liver cells, is one of the wonders of human physiology [1,2].

But even this tough organ has its limits. Chronic insults—whether from viruses, alcohol, autoimmune attacks, or metabolic stress—can overwhelm its repair mechanisms. Over time, scar tissue builds up, leading to fibrosis, cirrhosis, and in severe cases, liver failure or cancer. Cirrhosis alone claimed over 1.4 million lives in 2019, a number that's climbed sharply over the past few decades. For patients with end-stage disease, liver transplantation remains the only definitive cure. Yet, with transplant waiting lists growing faster than donor availability, and long-term outcomes still complicated by rejection and immunosuppression, it's clear we need better alternatives [[3], [4], [5]].

That's where tissue engineering steps in. Over the last couple of decades, researchers have turned to biomaterial scaffolds designed to mimic the liver's native extracellular matrix (ECM)—not just as structural support, but as a dynamic environment that guides cell behavior. Among the most promising of these scaffolds is the decellularized liver extracellular matrix (dLECM). By stripping away cells while preserving the complex network of collagen, fibronectin, growth factors, and other bioactive molecules, dLECM keeps the “biological blueprint” of the liver intact [6,7].

First introduced in rats by Uygun and colleagues using a perfusion-based method, dLECM has since been adapted across species and models [8]. It can be used as a whole scaffold—either implanted directly or repopulated with hepatocytes, stem cells, or progenitor cells—or processed further into an injectable hydrogel. This hydrogel form is especially attractive: it can be delivered minimally invasively, conforms to irregular tissue defects, and provides a 3D microenvironment that supports cell attachment, survival, and function [9].

In fact, over the past ten years, more than 30 studies have explored dLECM-based hydrogels in liver regeneration. Results show these hydrogels can help maintain hepatocyte function in culture, boost angiogenesis, reduce fibrosis, and even support the formation of organoid-like structures. They're not just scaffolds—they're active participants in healing [[10], [11], [12]].

Despite this growing body of work, there's been no systematic effort to pull all this knowledge together—until now. To our knowledge, this is the first systematic review focused exclusively on dLECM-derived hydrogels. We walk through the key steps: how liver tissue is decellularized, how the matrix is turned into a hydrogel, and how these materials are characterized and applied in both lab and animal models. We also highlight the current challenges—standardization, scalability, immune response—and point toward future directions that could move this technology closer to the clinic.

## 2 Method

### 2.1 Protocol and registration

The aim of this systematic review is to comprehensively evaluate the current knowledge on dLECM-based hydrogels, focusing on their preparation methods, characterization techniques, and applications in tissue engineering, particularly for liver regeneration. This review adheres to the PRISMA (Preferred Reporting Items for Systematic Reviews and Meta-Analyses) guidelines to ensure transparency and methodological rigor.

### 2.2 Search strategy

**Fig. 1:**
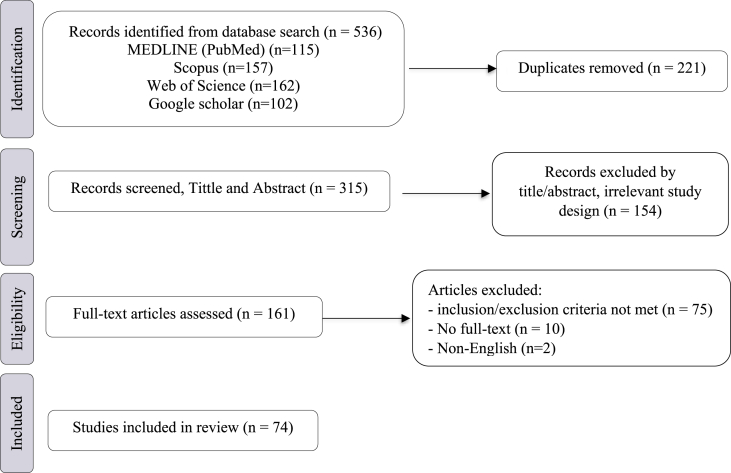
Study selection flow chart for studies in the systematic review.

The search strategy was designed based on the PICO framework (Population, Intervention, Comparison, and Outcome). A systematic literature search was conducted across four electronic databases: Scopus, MEDLINE (PubMed), Web of Science and Google Scholar up to July 18, 2025. Relevant terms related to related to liver decellularization, ECM, and hydrogels were used to construct the search query. The detailed search strategy is outlined in Fig. 1.

The final search query was structured as follows:

(Liver OR hepatic) AND ((Decellularized scaffold) OR (Decellularized Extracellular Matrix) OR (Decellularized ECM) OR (Decellularized ECMs) OR (Decellularized Extracellular Matrices) OR (decellulariz∗) OR (Acellularized Extracellular Matrix) OR (Acellularized ECM) OR (Acellular matrix)) AND ((Hydrogel) OR (gel) OR (gels) OR (In Situ Hydrogel) OR (Hydrogel, In Situ) OR (Patterned Hydrogel) OR (Hydrogel, Patterned) OR (injectable))

### 2.3 Eligibility criteria

Studies were included if they met the following criteria:

. Biomaterials specifically designed for liver tissue engineering applications.
. Investigated the preparation, physicochemical characterization, and biological applications of dLECM hydrogels in promoting hepatic cell viability, function, and liver regeneration.
. Included studies that combined dLECM hydrogels with other natural or synthetic hydrogels, growth factors, or cells (e.g., hepatocytes, stem cells) to enhance liver-specific tissue engineering outcomes.

Exclusion criteria:

. Reviews, case studies, book chapters, and conference articles.
. Studies not published in English.
. Articles without full-text availability.

No date restrictions were applied.

### 2.4 Study selection and data extraction

After conducting the initial search, a total of 536 articles were identified. Following the removal of duplicates (221 articles), 315 unique articles remained. These articles underwent a two-step screening process: first, titles and abstracts were reviewed for relevance. After the first round of screening, 161 studies were included for full-text screening. After the full-text screening, 87 studies were excluded for several reasons, and 74 studies were included for review, followed by a full-text assessment of potentially eligible studies. Ultimately, as depicted in Fig. 1 articles met the inclusion criteria and were included in this systematic review. Disagreements during the selection process were resolved through consensus discussions among the review team. Two authors independently screened papers based on the eligibility criteria mentioned before. Any discrepancies between selected studies from both authors were solved by the third author. Three authors independently extracted the data from eligible studies. Any discrepancies were solved by the third author. All data were recorded in Microsoft Excel for further reviewing.

### 2.5 Data extraction

Data were extracted using a standardized form designed to capture key information from each study. Extracted information included:

• Study details (author, year, country, tissue source)
• Decellularization method (technique, detergents, duration)
• Hydrogel fabrication process (lyophilization, digestion, neutralization, crosslinking)
• Characterization methods (histology, biochemical assays, rheology, SEM/TEM)
• Functional outcomes (cell viability, albumin secretion, urea synthesis, *in vivo* regeneration)

Two reviewers independently performed data extraction, and discrepancies were resolved through discussion or arbitration by a third reviewer.

### 2.6 Quality assessment

The quality of the included studies was assessed using appropriate tools, such as the Cochrane Risk of Bias Tool for randomized trials and the Newcastle-Ottawa Scale for observational studies. This assessment aimed to identify potential biases that could affect the validity of the findings.

### 2.7 Data synthesis

Data synthesis will be conducted through a combination of narrative synthesis and structured tabular summaries to provide a comprehensive overview of the current evidence on dLECM-derived hydrogels. The extracted data from the 74 included studies will be organized thematically to highlight key trends, methodological variations, and translational outcomes. First, the preparation methods of dLECM hydrogels will be systematically described, including tissue source (e.g., porcine, rat, human), decellularization techniques (immersion/agitation vs. perfusion, specific detergents such as SDS, Triton X-100, or ammonium hydroxide), physical processing steps (e.g., lyophilization, homogenization, pepsin digestion), and hydrogel formation strategies (e.g., thermal gelation, photocrosslinking, chemical crosslinkers like genipin or PEG-maleimide). Second, an overview of the characterization techniques employed across studies will be presented, encompassing histological (H&E, DAPI, IHC), biochemical (DNA, GAG, collagen content), ultrastructural (SEM, TEM), mechanical (rheology, compressive modulus), and functional assessments (gelation time, swelling ratio, biodegradability). Third, the applications of dLECM hydrogels in tissue engineering will be summarized, with emphasis on their use in 3D cell culture, organoid development, bioprinting, drug screening, and *in vivo* regeneration models, particularly in liver-specific contexts such as hepatocyte function, albumin secretion, CYP450 activity, and disease modeling. Finally, a comparative analysis will be performed to evaluate the impact of different decellularization protocols and hydrogel formulations on biological performance, including cell viability, differentiation, and regenerative outcomes. This comparative approach will also consider hybrid systems (e.g., dLECM combined with GelMA, chitosan, or PVA) and their advantages over pure dLECM or synthetic hydrogels. Together, this synthesis will enable a critical evaluation of the state-of-the-art in dLECM-based biomaterials and identify optimal practices for future research and clinical translation.

## 3 Result

The initial database search yielded 536 records. After the removal of 221 duplicates, 315 articles were then assessed by the title and the abstract. This initial screening resulted in the removal of 154 studies that were found to be irrelevant, leaving 161 articles that underwent detailed full-text evaluation. After detailed scrutiny of the full text, a further 87 studies were excluded for not meeting the criteria of eligibility. This process, including the inclusion of three supplementary articles, resulted in the final collection of 74 studies that were included in the present systematic review.

### 3.1 Physicochemical properties of synthesized hydrogels

Several analytical methods were utilized by the researchers to assess the efficacy of decellularization, integrity of the ECM components, and the physicochemical properties of the final dLECM hydrogels.Globally summarized results can be seen in Table 1.

**Table 1:** Study Characteristics, Cellular Applications, and Functional Outcomes of dLECM-Based Hydrogels.

Author (Year)	Decellularization Efficacy (DNA Quantification, Histology)	ECM Preservation (Collagen, GAGs, IHC)	Microstructure (SEM/TEM)	Mechanical Properties (Storage Modulus G′, etc.)	Physicochemical Properties (Gelation, Swelling, Biodegradability)
Agarwal, T (2018) [26]	DNA: 1.285 ± 0.497 % of native tissue.	Collagen: Not specified. GAGs:Significant reduction.	SEM: Showed characteristic banding pattern of the collagen nanofibrils.	Rheology: Yes.	Gelation Time: ∼20 min at 37 °C.
Agarwal, T (2019) [27]	DNA: ∼71.5 ± 5.26 ng/mg dry weight. H&E: Absence of observable cellular components.	Collagen: Not specified. GAGs:∼68.78 % retained.	SEM: Porous nature with interconnected pores.	Storage Modulus (G′): Yes.	Swelling:Yes. Biodegradability:Yes. Immunogenicity:Yes, tested.
Ahmed, E (2020) [28]	DNA: <5 % of native DNA retained. IHC:Staining for Ki67 performed.	Collagen: High amounts retained with no significant difference. IHC:Higher expression of albumin in treated groups.	SEM: Showed multiple areas of destruction.	Not specified.	Gelation Time:Within 1 h.
Almalla, A (2023) [29]	DNA: 270 ± 37 ng/mg.	Collagen: Constituted ∼97 % of the total detected matrisome. GAGs: sGAG content decreased significantly.	SEM: Yes.	Rheology: Yes. Storage Modulus (G′): Yes.	Gelation Time (t_50_):8.5 ± 4.2 min (Papain) vs. 11.6 ± 2.1 min (dECM-Pepsin).
Almalla, A (2024) [30]	DNA: Residual dsDNA content indicated efficient decellularization.	GAGs: Native sGAG content was conserved.	SEM: Yes.	Rheology: Yes. Storage Modulus (G′): Yes.	Swelling: Yes. Biodegradability:Faster enzymatic biodegradation in dECM-GMA.
B. Wang (2018) [15]	DNA: 98.9 % reduction.	Collagen: Retained ∼71 % of the total protein. GAGs:Retained ∼85 % of GAGs.	SEM: Yes.	Not specified.	Gelation: Occurred only at 5 and 10 mg/mL concentrations. Biodegradability:Yes, ∼52 % protein retained after 6h.
B. Wang (2020) [31]	DNA: ∼96.5 % reduction (∼111 ng/mg). IHC:Staining for α-Gal, fibronectin, laminin, type I & IV collagen performed.	Collagen & GAGs:Content was lower than native tissue but was higher in purified vs. non-purified ECM.	SEM: Yes.	Rheology: Yes. Storage Modulus (G′): Yes. Other:Thermal behavior (TGA).	Swelling (Water Absorption): Yes. Biodegradability:Yes.
Beachley, V (2018) [32]	Not specified.	Not specified.	Not specified.	Not specified.	Not specified.
Bhatt, S (2025) [22]	DNA: Significant reduction (p ≤ 0.05). H&E/DAPI:Confirmed minimal/absent nuclear material and preserved ECM architecture.	Collagen: Retained post-decellularization. GAGs: Increased post-decellularization. IHC: HepG2 cells expressed Albumin and Cytokeratin-18.	SEM: Porous structure with interconnected pores, favorable for cell infiltration and nutrient diffusion.	Rheology: Yes, showed shear-thinning behavior. Storage Modulus (G′): Highest in PVA/CH + dLM-FT.	Gelation: Achieved after one freeze-thaw cycle. Swelling:Increased up to 72 h, plateaued afterward. Biodegradability:Yes.
Biswas, S (2022)(33)	DNA: Reduced from 2800 ± 1.2 to 69 ± 0.3 ng/μL. H&E: Confirmed complete removal of cells.	Proteomics: Identified 149 proteins including collagen, laminin, fibronectin.	SEM/TEM: Showed a dense, interwoven fibrillar mesh with fibril diameters of ∼200–500 nm.	Rheology: Yes. Storage Modulus (G′): 2.8–4.0 kPa for the hybrid matrix (pure dipeptide gel was 7.2 kPa).	Biodegradability: Proteolytically stable due to the inclusion of a conformationally restricted dipeptide (IAF).
Bobrova, M. M. (2021) [34]	DNA: <1 % of native tissue; no DNA fragments >200 bp. H&E: Matrix architecture was preserved, no nuclear staining.	Histology: Masson's trichrome confirmed preserved skin layer structure in wound healing model.	SEM: Sinuous, rough topography with a highly nanoporous, branched pore system.	Not specified.	Biodegradability:Yes.
Bual, R. P. (2019) [35]	Not specified.	Total Protein: 43 % higher in L-ECM II vs L-ECM I. Collagen:61 % higher in L-ECM II.	SEM: Denser fiber network and larger fiber diameter in L-ECM II.	Rheology: Yes. Storage Modulus (G′): Yes. Mechanical Tests:Higher compression strength in L-ECM II gel.	Not specified.
Chen, J. (2023) [4]	H&E: Cell components were completely removed, leaving an empty honeycomb structure.	GAGs: Inapparent differences between native and dECM (10–15 ng/mL). IHC:Basement membrane proteins (collagen I, IV, laminin, fibronectin) retained.	SEM: Yes.	Not specified.	Not specified.
Coronado, R. E. (2017) [36]	DNA: Significant reduction. H&E:Complete nuclear material removal confirmed.	Collagen: Better preserved in Method B (NaDOC). GAGs:Preserved in both methods.	Not specified.	Not specified.	Immunogenicity:DNA quantification used as a proxy.
Coronado, R. E. (2019) [37]	Not specified.	Not specified.	Not specified.	Not specified.	Not specified.
D. S. Sun (2018) [38]	DNA: 290.67 ± 54.31 ng.	IHC: Types I and IV collagen and fibronectin were preserved.	SEM: Yes.	Not specified.	Swelling: Yes.
D. Y. Zhang (2015) [39]	DNA: 7.27 % remained after DNase vs 39.84 % with DI water wash. H&E/DAPI: Yes.	Not specified.	Not specified.	Not specified.	Gelation: No.
D. Udagawa (2024) [11]	H&E: Revealed transplanted cells within the L-ECM gel *in vivo*.	Proteomics: Yes.	SEM: Yes.	Rheology: Yes. Storage Modulus (G′): Yes.	Not specified.
Damania, A. (2017) [14]	DNA: <3–4 % of native tissue. H&E/DAPI: Little presence of nuclei.	GAGs: Higher GAG content per gram of matrix compared to native tissue.	SEM: Porous network with increased roughness upon dECM coating.	Not specified.	Immunogenicity:Yes.
Deegan, D. B. (2015) [40]	DNA: Below detection levels (<1 ng/mL). DAPI:Showed significant increase in cell attachment with increased ECM gel stiffness.	GAGs: 102.85 ± 16.92 μg/mg.	SEM: Porous network of the liver matrix.	Rheology: Yes. Storage Modulus (G′): Yes.	Immunogenicity:Yes.
Di Gravina, G. M. (2023) [41]	Not specified.	Not specified.	Not specified.	Rheology: Yes. Storage Modulus (G′): Yes.	Swelling: Yes. Biodegradability:Yes. Immunogenicity:Yes.
Dziki, J. L. (2017) [42]	Not specified.	Proteomics: Yes, SDS-PAGE analysis performed.	Not specified.	Not specified.	Not specified.
Elomaa, L. (2020) [43]	Not specified.	Not specified.	Not specified.	Rheology: Yes. Storage Modulus (G′): Yes.	Swelling: Yes. Biodegradability:Not specified. Immunogenicity:Yes.
G. S. van Tienderen (2023) [44]	Histology: No H&E, IHC, or DAPI data reported to confirm decellularization efficacy.	Not specified.	Not specified.	Not specified.	Not specified.
Gao, Y. (2023) [45]	DAPI: Confirmed cell viability and nuclear presence on scaffolds.	Collagen & GAGs:Preserved in dECM coating.	SEM: Assessed scaffold morphology and cell alignment.	Not specified.	Gelation: Not applicable (used as a coating).
Guagliano, G. (2022) [46]	DNA: 0.6 ng/μg residual DNA.	IHC: Confirmed preservation and distribution of ECM compounds.	Not specified.	Rheology: Yes. Storage Modulus (G′): Yes.	Gelation: Immediate gelation reported.
H. Ijima (2018) [47]	DNA: 92.4 % removed.	GAGs: 0.15 μg/mg in ECM solution. IHC:Collagen types I, III, IV, V, and laminin detected.	SEM: Yes.	Rheology: Yes, including distortion, frequency, and temperature dispersion. Storage Modulus (G′):Yes.	Gelation Time: 30 min. Biodegradability:Yes.
Hou, Y. T. (2020) [48]	Not specified.	GAGs: 3.8 ± 0.31 μg/mg of dry weight.	SEM: Yes.	Not specified.	Not specified.
Hussein, K. H. (2020) [49]	DNA: 9.49 ± 6.24 ng/mg of dry weight. H&E: Absence of nuclei or cytoplasm.	Collagen: 12.3 ± 0.89 mg/mg of dry weight. GAGs: 3.8 ± 0.31 μg/mg of dry weight.	SEM: Porous network of woven collagen fibers.	Not specified.	Gelation: Time to 50 % gelation (t_1_/_2_) was 39.16 ± 3.14 min. Swelling, Biodegradability, Immunogenicity:Yes.
Ijima, H. (2019) [50]	Not specified.	GAGs: 0.14 ng/mg-wet weight in decellularized liver (vs 0.53 in native).	SEM: Showed spherical morphology of hepatocytes embedded in a fiber-like skeletal structure.	Not specified.	Not specified.
J. S. Lee (2019) [51]	DNA: >99.5 % removed.	Collagen: IHC showed collagen I was preserved. GAGs: No significant difference before and after decellularization.	SEM: Showed nanofibrillar internal structures in the hydrogel.	Rheology: Yes.	Biodegradability:Yes.
Jakus, A. E. (2017) [52]	H&E: Cell nuclei were not observed in decellularized tissues.	Not specified.	SEM: Showed conserved and varied structures of the six tissue ECMs.	Not specified.	Biodegradability:Yes.
Jin, Y. (2018) [53]	DNA: 9.7 % of cells remained (as per source). H&E:Absence of cellular components.	GAGs: 78.7 % remained.	SEM: Showed the presence of nanofibrillar internal structures.	Rheology: Yes.	Immunogenicity:Yes.
Jin, Y. Y. (2023) [54]	Not specified.	Not specified.	SEM: Showed a porous dECM hydrogel with no residual cells.	Storage Modulus (G′): Yes.	Immunogenicity:Yes.
Kellaway, S. C. (2023) [55]	DNA: >90 % reduction. H&E:Lack of observable nuclei.	Collagen: Abundant collagen fibrils observed via Picrosirius Red. GAGs: sGAG content: SIS-ECM > LIV-ECM > B-ECM.	Not specified.	Rheology: Yes.	Gelation: Gelation profile was observed and kinetics recorded but results not detailed.
Khati, V (2022) [20]	DNA: 98.6 % reduction (32.1 ng/mg). H&E:Showed removal of cell debris.	Collagen: Increased to 29.05 μg/mg. GAGs: 74 % reduction.	Not specified.	Rheology: Yes. Viscosity increased 16-fold and storage modulus 32-fold with crosslinking.	Gelation: Immediate gelation; crosslinked in 30 min.
Khati, V (2022) [56]	Not specified.	Collagen: Used as a control group.	SEM: Yes.	Rheology: Yes.	Gelation: Increased complex modulus and faster gelation in dLM-PEG-T vs. dLM-PEG.
Kim, M. (2023) [57]	DNA: 96.13 % removed. H&E:Most cellular components were removed. DAPI:Performed as part of immunofluorescence.	Collagen: 441.84 % remained (concentration increased). GAGs:55.67 % remained.	Not specified.	Rheology: Yes. Viscosity of bio-inks increased with higher gelatin and dECM concentration.	Not specified.
Kim, M.K. (2023) [58]	Not specified.	Collagen & GAGs:Preserved in dECM.	SEM: Images showed microstructure of bioinks.	Rheology: Yes. Compressive Modulus:Significantly improved in dECM-gBioink. Storage Modulus (G′): Yes.	Gelation: Achieved via thermal and physical crosslinking.
Kojima, H. (2023) [59]	DNA: >99.5 % reduction. H&E: No cellular components, well-preserved scaffold.	Collagen: No difference between dECM and native tissue (7.4 vs 7.2 μg/mg). GAGs:Slightly reduced (1.7 vs 1.1 μg/mg).	SEM: Yes.	Rheology: Yes. Storage Modulus (G′): Yes.	Gelation: Occurred at ≥4 mg/mL, stable and solidified. Time to Gelation: ∼30 min.
Lee, J. (2014) [21]	DNA: 2.8 % of native. H&E/DAPI:Confirmed removal of cell components/nuclei.	Collagen: More native-like nanofibrillar architecture vs. Col I. GAGs: 83.8 % of native retained.	SEM:Nanofibrillar porous collagen structure.	Rheology: Yes. Storage Modulus (G′): Frequency-independent, stable gel behavior.	Gelation Time: 4–9 min, faster at higher concentration.
Lewis, P. (2018) [60]	DNA: ∼1 % of native liver.	Collagen: Content increased. GAGs:Content reduced. IHC: Staining for SOX9, HNF1β, E-cadherin, etc. performed.	SEM: Yes. TEM: Yes, showed formation of apical microvilli.	Rheology: Yes. Storage Modulus (G′): Yes.	Gelation Time:Under 10 min.
Lewis, P. (2019) [61]	Not specified.	Collagen: Number of assembled collagens via SHG microscopy decreases as strut width decreases.	SEM: Showed reduced porosity in 1–2 mm struts and larger pores in 4–8 mm struts.	Rheology: Yes, final storage and loss moduli were identical regardless of temperature ramping. Storage Modulus (G′):Yes.	Not specified.
Li, S. (2021) [16]	DNA: 2.2 % of native tissue. H&E:Confirmed absence of nuclei.	IHC: Staining for collagen I, fibronectin, laminin performed. GAGs: Content lower in acellular vs. native.	SEM: Showed nanofibrous scaffolds with interconnecting pores.	Not specified.	Not specified.
Loneker, A. E. (2016) [19]	DNA: Ranged from 98 ng/mg (porcine) to 641 ng/mg (human). H&E: No basophilic nuclear staining.	Collagen: Each species had distinct collagen architecture. GAGs: Ranged from 583 μg/g (porcine) to 10,907 μg/g (human). Proteomics: Yes.	SEM: Denser fiber network in porcine vs. canine ECM.	Rheology: Yes. Storage Modulus (G′): Yes.	Gelation: Time to 50 % gelation ranged from <5 min (rat) to >10 min (human).
Lu, S. (2018) [17]	DNA: 21.99 ng/mg. H&E/DAPI:Confirmed absence of cells/nuclei.	Collagen: 18-fold increase in dry concentration. GAGs:2.5-fold increase. IHC: Positive for Coll I/IV, fibronectin, laminin.	SEM: Mesh-like structure.	Young's Modulus:No significant difference among hybrid hydrogels.	Not specified.
M. Tabuchi (2015) [62]	Not specified.	Not specified.	SEM:Performed on powder.	Not specified.	Not specified.
Ma, X. (2018) [14]	DNA: <50 ng/mg. H&E: Absence of nuclear staining.	Collagen: Enriched in dECM relative to native. GAGs: 30 % of native. IHC: Positive for collagen I/IV, fibronectin, laminin.	SEM:Preservation of intact collagen fibrils.	Not specified.	Not specified.
Milton, L. (2024) [63]	H&E/DAPI:Decrease of nuclei in dECM.	GAGs: High retention in Triton X-100 protocols. Proteomics: Yes, protein count varied with decellularization protocol.	Not specified.	Young's Modulus:Tunable between ∼1 and 7 kPa.	Swelling: Yes. Other: Crosslinking time analysis.
Nakamura, S. (2014) [64]	Not specified.	Histology: Coomassie blue staining of gels performed.	Not specified.	Not specified.	Not specified.
Nishiguchi, A. (2021) [65]	Not specified.	Not specified.	Not specified.	Not specified.	Not specified.
Prebeg, T. (2023) [66]	Microscopy: SLS detergents caused tissue damage; SLES showed better preservation.	Not specified.	Not specified.	Not specified.	Gelation Time: 30–60 min depending on detergent concentration and wash time.
Rajabi, S. (2020) [67]	DNA: Dramatically reduced (98 % decrease). H&E/DAPI:Confirmed absence of cell nuclei.	Collagen & GAGs:Retained (Masson's Trichrome & Alcian Blue). IHC: ECM proteins were well-preserved.	Not specified.	Not specified.	Gelation: Warmed to 37 °C for 10 min to form final hydrogel.
Ravichandran, A. (2021) [68]	DNA: 2.5 to 5-fold drop after day 1. H&E: Loss of native lobular morphology, no visible nuclei.	IHC: ECM collagen was preserved.	Not specified.	Compressive Modulus:Increased with increasing crosslinking time.	Gelation: Protocol 2 gelled in 30 min; Protocol 1 did not gel after 6h. Swelling: Yes.
Saheli, M. (2018) [13]	DNA: 2.9 % of normal tissue. H&E/DAPI:Confirmed no intact cells/nuclei.	Collagen: Preserved (Masson's Trichrome). GAGs: 75 % remained intact. IHC:Immunofluorescence confirmed ECM glycoproteins.	SEM: Pore size of 382 ± 71 μm in LEM gel.	Rheology: Yes. Loss Moduli: 7.3 Pa (gel) vs 1.19 Pa (solubilized).	Gelation: Gel found 5 min past neutralization; incubated for 1h to form gel.
Sarika, N. (2020) [69]	DNA: 0.9–19.6 ng/mg ECM.	Collagen: 12 collagen types found (23 % of total proteins). Proteomics: Yes, 52 proteins identified.	Not specified.	Not specified.	Not specified.
Sasikumar, S (2022)(70)	DNA: 4.85 % of native tissue retained (5.17 ng/mg). H&E: Confirmed absence of nuclei.	Collagen: 59.48 % retained. GAGs: 61.25 % retained.	SEM: Showed a highly compact fibrous network compared to native tissue.	Not specified.	Swelling: Showed minimal deswelling (0.65 %–2.45 %) over 30 days. Porosity: ∼90 %, stable over 32 days.
Serna-Márquez, N. (2020) [71]	Not specified.	Histology: Silver staining and Coomassie staining performed.	Not specified.	Elastic Moduli:Tested on PAA hydrogels (1 kPa and 20 kPa).	Not specified.
Skardal, A. (2012) [72]	IHC: Strong presence of cytoplasmic albumin.	Collagen: 91.33 μg/mL in LEE. GAGs: 86.00 μg/mL in LEE.	Not specified.	Not specified.	Not specified.
Skardal, A. (2015)	Not specified.	Collagen & GAGs:Analyzed in ECM solution.	Not specified.	Rheology: Yes. Storage Modulus (G′): Yes.	Not specified.
Tabatabaei Rezaei, N (2024) [73]	DAPI: Confirmed cell viability and nuclear presence.	Collagen & GAGs:Preserved in dECM.	SEM: Assessed hydrogel microstructure.	Rheology: Yes. Compressive Modulus:Enhanced. Storage Modulus (G′):Improved with LdECMMA.	Gelation: Achieved within 1 min. Swelling & Biodegradability:Yes.
Tabatabaei Rezaei, N (2025) [25]	DNA: <2.7 % of native liver. H&E/DAPI: No nuclei observed.	Collagen: Preserved (Masson's Trichrome). IHC: Higher albumin in LdMA groups.	SEM: Pore size decreased with LdMA (100.3 → 29.1 μm).	Rheology: Yes, showed shear-thinning. Compressive Modulus: Tunable (2.06–36.21 kPa). Storage Modulus (G′): Increased with LdMA.	Gelation Time: <1s–30s (phototheology). Swelling Ratio:Decreased with LdMA (2302 %→1370 %). Biodegradability:Slower with LdMA (>48h).
Takeda, Y. (2014) [74]	Not specified.	Not specified.	SEM: Yes.	Young's Modulus:Measured on 2D films.	Not specified.
W. Jeong (2021) [75]	DNA: Removal efficiency: SDS > TXA > SDC > TX.	Collagen & GAGs:Assessed on powder; SDS and SDC caused severe damage.	SEM: Yes.	Rheology: Yes, including temperature sweep analysis. Storage Modulus (G′):Yes.	Swelling: Yes.
Willemse, J. (2022) [23]	Not specified.	Collagen & GAGs:No difference between human and porcine hydrogels. IHC:Positive for Collagen I, III, IV.	SEM: Yes.	Rheology: Yes. Nano-indentation:Yes. Storage Modulus (G′):Yes.	Gelation Kinetics:Turbidity assay showed longer lag-phase for PLECM. Swelling: PLECM ∼40 %, HLECM ∼33 %.
Wolf, M. T. (2012)(76)	DNA: 94.7 % reduction. H&E: Confirmed absence of nuclei.	Collagen: 60 % retained. GAGs: 30 % retained.	SEM: Showed nanofibrous structure.	Rheology: Yes. Storage Modulus (G′): 100–200 Pa	Gelation Time: 20–40 min.
X. Zhang (2015) [39]	DNA: 3 % of native tissue (0.48 vs 15.4 ng/mg).	Not specified.	Not specified.	Not specified.	Not specified.
Xinyi Li (2025) [77]	Histology: γ-H2AX staining showed significant increase in expression.	Not specified.	Not specified.	Not specified.	Not applicable.
Xu, Z. (2025)(78)	DNA: Extraction used for proteomic analysis. Histology:Showed presence of donor cells in grafts.	Collagen & GAGs:Preserved in dECM. IHC: Confirmed proliferation and differentiation markers.	Not specified.	Rheology: Yes, viscoelastic properties enhanced by THBS1. Storage Modulus (G′):Yes.	Not specified.
Y. T. He (2020) [79]	DNA: 1.04 % of fresh liver.	IHC: Retained collagen I, IV, fibronectin, and laminin.	SEM: Yes.	Not specified.	Not specified.
You, P. Y. (2024) [80]	DNA: 21.74 ± 3.4 ng/mg residual DNA.	Collagen & GAGs:Active components retained.	SEM: Yes.	Rheology: Yes. Storage Modulus (G′): Yes.	Swelling & Biodegradability:Yes.
Yu, C. (2019) [18]	DNA: <50 ng/mg dry tissue.	IHC: Positive for Collagen I, IV, laminin, fibronectin.	SEM: Yes.	Not specified.	Swelling: Yes.
Zhang, J. (2025) [81]	H&E/PAS/Sirius Red: Showed improved tissue repair, glycogen production, and collagen deposition in treated groups.	IHC: CD31^+^ vessels and CK19+ bile ducts comparable to normal liver. Proteomics:Performed on EVs, not dECM.	SEM: Images of 3D-printed scaffolds. TEM: Used for EV characterization.	Not specified.	Biodegradability:Negligible degradation after 24h. Immunogenicity:Reduced pro-inflammatory cytokines.
Zou, C. Y. (2025) [24]	H&E: Showed hydrogel sealing wounds, cell infiltration, and tissue regeneration *in vivo*.	Histology: Sirius red confirmed collagen deposition *in vivo*.	SEM: Showed powder morphology and hydrogel microstructure.	Rheology: Yes. Storage Modulus (G′): 5157 Pa for APSO hydrogel.	Gelation Time: 5 s. Swelling Rate: 69.09 %. Biodegradability:Complete in 28 days *in vivo*.

#### 3.1.1 Verification of decellularization and ECM preservation

Histologic studies, especially Hematoxylin and Eosin (H&E) and DAPI staining, confirmed the extrusion of nuclear and cellular material [13,14]. Detailed biochemical studies determining the amount of DNA confirmed these results, the bulk of the samples under 50 ng per mg dry ECM, and some with over a 98 % clearance of the DNA [15,16].

Furthermore, quantitative chemical assays, including immunohistochemistry (IHC) as well, confirmed the preservation of distinctive ECM proteins. Matrical components like collagen types I, III, and IV, laminin, and fibronectin were preserved [17,18]. Collagen concentrations tended to be in the range between 40 and 200 μg/mg, whereas GAGs were found at concentrations between 30 and 80 μg/mg. These levels tended to change with the extent of the decellularization process [19,20].

#### 3.1.2 Microstructure and mechanical properties

Scanning electron microscopy (SEM) showed a very porous, fibrous connective network, whose pore size was between 50 and 200 μm, which is suitable for transportation of nutrients and migration of cells [21,22].

Mechanical tests on 40 validated the soft, viscoelastic nature of dLECM hydrogels. Rheological data always showed storage modulus (G′) values higher than loss modulus (G″) values, typical stable gel behavior. G′ values quoted ranged between ∼100 Pa and in excess of 5000 Pa and depended on the concentration of the ECM (usually 5–20 mg/mL) and the crosslinking method [23,24]. Most compositions of hydrogels showed the desirable property of shearthinning, beneficial in the extrusion-based 3D bioprinting context [25].

#### 3.1.3 Gelation dynamics and related properties

Gelation was brought about almost entirely by thermal self-assembly at 37 °C, and the time interval before the commencement of gelation varied between 10 min and 1 h [19,21]. Turbide measurements indicated a concentration-dependent initial lag followed by the formation of increasing turbide, characteristic of collagen fibrillogenesis [23]. Other physicochemical properties, including swelling ratios and *in vitro* degradation times, were investigated as well, and sample data are tabulated in Table 1.

### 3.2 Fabrication, formulation, and crosslinking strategies for the dLECM

The methods utilized in the creation of dLECM-derived hydrogels, although generally similar, showed significant differences among the 74 articles surveyed. Key differences were seen with respect to the origin of the tissue, the methods adopted in decellularization, as well as the end composition and crosslinking approaches utilized in the construction of viable biomaterials, as summarized in Table 2.

**Table 2:** Summary of Decellularization and Hydrogel Fabrication Methods in dLECM-Based Studies.

Author (Year, Country)	Tissue Source & Pre-Processing	Decellularization Method (Technique, Detergents, Duration)	dECM Solubilization (Enzyme, Acid, Duration)	Hydrogel Preparation Process	Final Formulation & Crosslinking
Agarwal, T (2018, India) [26]	Source: Others (Caprine). Pre-Processing:Chopped into pieces, washed with sterile HBSS.	Technique:Immersion/Agitation. Detergents: 2 % Triton-X100, 0.05 mM EDTA, and 0.025 % ammonium hydroxide.	Enzyme/Acid:Pepsin in 0.01 M HCl to a final concentration of 12 mg/mL. Duration: 48 h at room temperature.	CLECM and collagen solutions mixed with 10 % (v/v) of 10 × PBS/sterile water, pH adjusted using 1 N NaOH, briefly centrifuged, and incubated at 37 °C for 20 min.	Method: Self-assembly. Details:Incubated at 37 °C for 20 min. Additives:Composite with Collagen.
Agarwal, T (2019, India) [27]	Source: Others (Caprine). Pre-Processing:Chopped into small cubes, agitated (∼200 rpm) for ∼72 h at 4 °C, washed, dialyzed, lyophilized.	Technique:Immersion/Agitation. Detergents: 2 % Triton-X100, 0.05 mM EDTA and 0.025 % ammonium hydroxide for ∼72 h at 4 °C.	Enzyme/Acid:10 % (w/w) pepsin in 0.01 M HCl to achieve a final concentration of 12 mg/mL.	Neutralized, frozen at −20 °C, lyophilized for 12 h, then re-neutralized and re-lyophilized.	Method: Chemical. Details: Not specified.
Ahmed, E (2020, South Korea) [28]	Source: Rat. Pre-Processing:Perfusions of 1 % Triton X-100/ammonium hydroxide mixture, then scaffolds washed with PBS for 2 h.	Technique:Immersion/Agitation. Detergents: 1 % Triton X-100/ammonium hydroxide mixture 0.1 %. Enzymes:DNase enzyme (30 μg/mL) for 2 h.	Enzyme/Acid:100 mg of pepsin in 90 mL of HCl (0.1 M).	Sterilized decellularized liver scaffolds were lyophilized, milled, mixed with pepsin and HCl, agitated, and neutralized by NaOH (1 N).	Method: Self-assembly. Details:Thermal gelation. Additives:Composite with silver nanoparticles (AgNPs).
Almalla, A (2023, Germany) [29]	Source: Porcine. Pre-Processing:Pooled slices washed in distilled H_2_O or PBS at 4 °C for 24 h, resuspended, and agitated for 2 h.	Technique:Immersion/Agitation. Detergents: 1 % Triton X-100 for 24 h.	Enzyme/Acid:Soluble dECM-Papain and dECM-Pepsin digests (2 mg/mL) in 0.01 M HCl.	Pregel solutions got solubilized and neutralized with distilled water.	Method: Self-assembly. Details:Thermal gelation.
Almalla, A (2024, Germany) [30]	Source: Porcine. Pre-Processing:Sectioned, resuspended in fresh medium, filtered, and washed gently for 30 min with distilled water.	Technique:Immersion/Agitation. Detergents: Agitated for 2 h, and replaced with 1 % Triton X-100 for another 24 h.	Enzyme/Acid:The dry dECM-digest (1 g) was solubilized in 100 mL of 0.01 M HCl.	The solubilized solution was neutralized with 10 M NaOH, dialyzed against distilled water, and lyophilized.	Method:Photocrosslinking. Additives: dECM was chemically modified with glycidyl methacrylate (GMA) to create a photocurable bioresin.
B. Wang (2018, USA) [15]	Source: Rat. Pre-Processing: Not reported.	Technique: Perfusion (Portal vein). Detergents:1 % Triton X-100, 0.1 % NH_4_OH (time not reported).	Enzyme/Acid:100 mg pepsin added to 90 mL of 0.1 M HCl, kept at a constant stir.	dECM was lyophilized, milled into powder, solubilized, and then neutralized.	Method: Self-assembly. Details:Gelled only at 5 and 10 mg/mL concentrations. Concentration: 1g initial dECM.
B. Wang (2020, USA) [31]	Source: Porcine. Pre-Processing:Deionized water perfusion for 3 h to remove blood.	Technique: Perfusion (Portal vein). Detergents:0.5 % SDS, 1 % Triton X-100, 0.1 % ammonium hydroxide (NH_4_OH) for 72 h.	Enzyme/Acid:100 mg of pepsin in 90 mL of 0.1 M HCl with constant stirring for 48 h.	dECM was sectioned, lyophilized, milled, solubilized, neutralized, purified with acetone, redissolved, and re-lyophilized into powder. ECM and gelatin powders were then mixed.	Method: Self-assembly. Additives: ECM and gelatin powders mixed at varying ratios (1E-1G to 3E-5G) to form a sponge. Concentration: 1g initial dECM.
Beachley, V (2018, USA) [32]	Source: Porcine. Pre-Processing:Pre-cut tissue was incubated in 3 % peracetic acid at 37 °C for 3 h to solubilize cytosolic components and remove nucleic acids.	Technique:Immersion/Agitation. Detergents: 1 % Triton X-100 solution containing 2 mM EDTA for 18 h.	Not specified.	No details provided.	Method: Not specified. Additives:Composite of ECM particles and glycosaminoglycans (GAGs).
Bhatt, S (2025, India) [22]	Source: Caprine (goat) liver. Pre-Processing: Tissue minced into fine cubes, washed with HBSS and PBS.	Technique: Chemical (Triton X-100) and enzymatic method. Detergents: 10 % Triton X-100 for 72 h.	Enzyme/Acid:Pepsin digestion in 0.5 M acetic acid for 48 h.	Decellularized liver tissue was lyophilized, pepsin-digested, neutralized, and blended with PVA/chitosan.	Method: Physical Crosslinking. Details: One freeze-thaw cycle was used for gelation. Additives:PVA/Chitosan composite hydrogel.
Bobrova, M. M. (2021, Russia) [34]	Source: Rat. Pre-Processing: Not specified.	Technique:Immersion/Agitation. Detergents: Three sequential stages of perfusion: (a) 0.1 % SDS in PBS with 1 % Triton X-100; (b) 0.1 % SDS in PBS with 2 % Triton X-100; (c) 0.1 % SDS in PBS with 3 % Triton X-100. Perfusion at 150 mL/h for 72 h.	Not specified.	Liver perfused with PBS/solution/PBS and stored at 4 °C until use.	Method: Self-assembly. Details:Thermal gelation.
Biswas, S (2022, India) [33]	Source: Rat. Pre-Processing: Portal vein cannulation.	Technique: Perfusion (Portal vein). Detergents: 1 % Triton-X 100 with 0.1 % ammonium hydroxide for 20–24h.	Enzyme/Acid: Pepsin (2 mg) in 0.5 M acetic acid (1 mL) at 37 °C until solubilized.	Dipeptide (IAF) dissolved in 1-propanol, sonicated. Solubilized liver ECM (sLEM) added to the dipeptide solution, then gelled by adding 0.8 M sodium acetate buffer (pH 7.2).	Method: Self-assembly. Additives: Hybrid formulation with a synthetic dipeptide (isoleucine-α,β-dehydrophenylalanine, IAF).
Bual, R. P. (2019, Philippines) [35]	Source: Rat. Pre-Processing: Sliced, soaked in Triton X-100, dialyzed, and lyophilized for 24 h.	Technique:Immersion/Agitation. Detergents: 1 % Triton X-100 at 4 °C for 3 days.	Enzyme/Acid:10 mg pepsin in 10 mL of 0.1 N HCl. The mixture was constantly stirred for 3 days at 4 °C.	No details provided.	Method: Self-assembly. Details:Incubation at 37 °C for 30 min.
Chen, J. (2023, China) [4]	Source: Rat. Pre-Processing: Liquid perfusion at a speed of 30 mL/min.	Technique: Perfusion (Portal vein). Detergents:1 % (v/v) Triton X-100 for 1h then 0.1 % (w/v) SDS for 1h, followed by washout.	Enzyme/Acid:Pepsin solution prepared by 0.5 M acetic acid to digest for 48 h at 37 °C.	Decellularization, washed with alcohol and PBS solution, freeze dissolved, neutralization, freeze. The lyophilized powder was added to a pre-prepared GelMA solution.	Method: Self-assembly. Additives: Gelatin methacryloyl (GelMA).
Coronado, R. E. (2017, USA) [36]	Source: Porcine liver. Pre-Processing: Tissue slicing and rinsing.	Technique: Not specified. Detergents: Method A: 3 % Triton X-100 for 1 h; Method B: 4 % Sodium deoxycholate (NaDOC) for 1 h.	Not applicable.	Porcine liver tissue was sliced and decellularized, followed by rinsing, sterilization, and ECM preservation for use as a substrate.	Not applicable (study focused on decellularization, not hydrogel formation).
Coronado, R. E. (2019, USA) [37]	Source: Porcine. Pre-Processing:Sliced, decellularized with mild agitation (100–125 rpm).	Technique:Immersion/Agitation. Detergents: 1 % Triton X-100 containing 0.1 % ammonium hydroxide for 8 h. Enzymes: 0.025 % Trypsin-EDTA at 37 °C for 1 h.	Enzyme/Acid:Acetic acid 0.001–1 M and porcine pepsin, in a 5:1:2 (mg:mL:mg) proportion, at 4 °C for 72 h.	Decellularized, solubilized, then the gel solution was filtered.	Method: Self-assembly. Details:Thermal gelation.
D. S. Sun (2018, China) [38]	Source: Porcine. Pre-Processing:Freeze-thawed, sliced into 5 mm-thick sheets, agitated at 150 rpm, washed with deionized water for 72h.	Technique:Immersion/Agitation. Detergents: 1 % (v/v) Triton X-100 and 0.1 % (v/v) ammonium hydroxide for 96h.	Not specified.	No details provided.	Method: Physical crosslinking. Details: Ionic crosslinking with calcium chloride. Additives: DLM powder combined with sodium alginate in concentrations of 0.5 %, 1 %, or 2 %.
D. Y. Zhang (2015, China) [39]	Source: Porcine. Pre-Processing:Cut into <1 cm^3^ cubes, frozen at −80 °C for 24h, lyophilized for 48h, and homogenized into particles for 3–30 min.	Technique:Immersion/Agitation. Detergents: Not specified. Enzymes:Three methods tested [1]: deionized water [2], 0.2 mg/mL DNase [3], 5 % fetal bovine serum (FBS, v/v).	Enzyme/Acid:100 mg pepsin in 45 mL of 0.1 N HCl.	Lyophilized tissue was ground, mixed with pepsin, sterilized by gamma irradiation, incubated in HCl for 48h, centrifuged, neutralized with NaOH, and stored at −80 °C.	Method: Chemical. Additives:Hyaluronic acid-based hydrogel decorated with heparin. Concentration: 1 g.
D. Udagawa (2024, Japan) [11]	Source: Porcine. Pre-Processing:Frozen at −80 °C, thawed at 4 °C for 24h, cut into small pieces, rinsed three times a day for 2 days at 4 °C with PBS under constant stirring.	Technique:Immersion/Agitation. Detergents: 1 % TritonX-100 and ammonium hydroxide for 5 days.	Not specified.	Lyophilized, acidified and solubilized, neutralized, then a second lyophilization, followed by gelation.	Method: Self-assembly. Details:Thermal gelation. Concentration:40 μl.
Damania, A. (2017, India) [14]	Source: Porcine. Pre-Processing:Sliced, soaked in 300 mL of Triton X-100 (1 %) and stirred at 4 °C for 4 days, washed with CMF-PBS for 24–48 h, then dialyzed.	Technique:Immersion/Agitation. Detergents: 1 % Triton X-100 for 4 days. Enzymes:DNase and RNase.	Enzyme/Acid:0.1 N HCl supplemented with 10 mg/mL pepsin for 72 h at 4 °C.	Decellularized, solubilized, dialyzed for 48 h at 4 °C, followed by washing.	Method: Chemical. Details: 1 % glutaraldehyde for 3 h at room temperature.
Deegan, D. B. (2015, USA) [40]	Source: Rat. Pre-Processing:Perfusion of Triton X-100 solutions, excision of liver, PBS washing.	Technique: Perfusion (IVC). Detergents: 300 mL each of 1 %, 2 %, and 3 % Triton X-100 solutions in PBS. Enzymes:DNeasy and miRNeasy kits used for nucleic acid removal.	Enzyme/Acid:0.1 N HCl and pepsin.	Decellularized, lyophilized, solubilized, centrifuged, and filtered with a 0.2 μm syringe filter.	Method: Chemical. Details:Crosslinked using 1 % glutaraldehyde for 3 h at room temperature.
Di Gravina, G. M. (2023, Italy) [41]	Source: Porcine. Pre-Processing:Cut into portions, transferred into 50 mL tubes, 12 h orbicular shaking (120 rpm), washed with sterile deionized H_2_O.	Technique:Immersion/Agitation. Detergents: 48 h of 0.15 % v/v SDS.	Enzyme/Acid:0.1 M HCl solution with a final concentration equal to 2 % w/v and digested with pepsin for 24 h at 37 °C.	The digested solution was frozen and lyophilized.	Method: Chemical. Details: Addition of a CaCl_2_-based solution at 60s after the start of rheological test.
Dziki, J. L. (2017, USA) [42]	Source: Porcine. Pre-Processing:Mechanical slicing/massage to aid cell lysis.	Technique:Immersion/Agitation. Detergents: 3 % Triton X-100, 4 % deoxycholate. Enzymes: 0.02 % trypsin/0.05 % EDTA.	Enzyme/Acid:100 mg of pepsin were mixed in 100 mL of 0.01 M HCl.	Scaffolds were lyophilized and milled to form a particulate powder, then solubilized with pepsin to yield a 10 mg/mL solution and neutralized.	Method: Self-assembly. Details:Thermal gelation. Concentration: 10 mg/mL.
Elomaa, L. (2020, Germany) [43]	Source: Rat. Pre-Processing:Perfused with PBS for 10 min.	Technique:Immersion/Agitation. Detergents: 1 % Triton X-100 for 90 min and 1 % SDS for 90 min, rinsed with PBS overnight.	Enzyme/Acid:Acidic pepsin solution (1 mg/mL in 0.01 N HCl) at room temperature for 3 days.	dECM was solubilized, neutralized, dialyzed against distilled water for 2 days, and then lyophilized.	Method:Photocrosslinking. Additives:GelMA/PCL and dECM/PCL resins.
G. S. van Tienderen (2023, Netherlands) [44]	Source: Human. Pre-Processing:Not specified.	Technique: Perfusion. Detergents: Continuous perfusion with Triton X-100 for 120 min followed by 9 cycles of perfusion with 10L Triton X-100 (120 min each). Enzymes:10 mg/L DNase type I for 8 h.	Enzyme/Acid:10 % (w/w) Pepsin in 0.5 M Acetic Acid over 72 h.	Lyophilized and pulverized, then digested, neutralized, and solubilized. If needed, solubilized HLECM was diluted to 8 mg/mL.	Method: Chemical. Details: Enzymatic crosslinking with H_2_O_2_. Additives:Dextran-tyramine and horseradish peroxidase. Concentration: 40 mg/mL.
Gao, Y. (2023, United Kingdom) [45]	Source: Porcine liver. Pre-Processing: Not specified.	Not specified.	Not specified.	dECM was used as a coating material on electrospun nanofibers to enhance hepatocyte functionality.	Not applicable (coating, not a hydrogel).
Guagliano, G. (2022, Italy) [46]	Source: Porcine. Pre-Processing:Cut into pieces, injected with decellularization buffer, agitated (∼140 rpm) for 72 h, washed with sterile DI water, frozen at −80 °C overnight, lyophilized.	Technique:Immersion/Agitation. Detergents: 1 % (w/v) SDS and 1 % (v/v) Triton X-100 in 1 M Tris-HCl buffer with pH 7.4.	Not specified.	Formed by sequentially mixing different solutions and suspensions with coupled syringes.	Method: Not specified. Details:Immediate gelation.
H. Ijima (2018, Japan) [47]	Source: Porcine. Pre-Processing:Cryopreserved at −80 °C, then sliced to 2 mm.	Technique:Immersion/Agitation. Detergents: 1 % Triton X-100.	Enzyme/Acid:1 mL of pepsin solution (1 mg/mL in 0.1 N HCl).	dLM was lyophilized, solubilized, then neutralized to pH 3.	Method: Self-assembly. Details:Thermal gelation. Concentration: 10 mg of lyophilized dECM.
Hou, Y. T. (2020, Taiwan) [48]	Source: Rat. Pre-Processing: PBS solution injected into the liver to wash off blood.	Technique: Perfusion (IVC). Detergents: 1 % Triton X-100 solution for 20 min, then 1 % SDS solution for 12 h, and PBS for 30 min.	Enzyme/Acid:Solubilized by 1 M HCl, stirred at room temperature for 5 h.	Solubilized, neutralized with Milli-Q water, stirred at room temperature for 15 min, washed, and freeze-dried for 2–3 days.	Method: Self-assembly. Additives: Gelatin-HPA-DLM hydrogel, Gelatin-HPA hydrogel.
Hussein, K. H. (2020, Egypt) [49]	Source: Mice. Pre-Processing:Infused with 0.1 % SDS and distilled water at a flow rate of 2 mL/min for 120 min, then perfused with PBS for 30 min.	Technique:Immersion/Agitation. Detergents: 0.1 % SDS for 120 min.	Enzyme/Acid:0.1 M HCl to make a 1 mg/mL pepsin solution for 72h.	Lyophilized, solubilized by pepsin, and neutralized.	Method: Self-assembly. Details:Thermal gelation.
Ijima, H. (2019, Japan) [50]	Source: Porcine. Pre-Processing:Cut into pieces, washed, organ perfused by CMF-PBS.	Technique:Immersion/Agitation. Detergents: 1 % Triton X-100 in CMF-PBS for 120 h.	Enzyme/Acid:1 mL of a pepsin solution (1 mg/mL in 0.1 N HCl) at 4 °C for 72 h.	Decellularized, lyophilized, minced, solubilized, neutralized, then the dialyzed L-ECM was incubated at 37 °C for 30 min.	Method: Self-assembly. Details:Thermal gelation.
J. S. Lee (2019, South Korea) [51]	Source: Porcine. Pre-Processing:Not specified.	Technique:Immersion/Agitation. Detergents: 1 % (v/v) Triton X-100 and 0.1 % ammonium hydroxide for 48h with agitation at 120 rpm.	Enzyme/Acid:pepsin 4 mg/mL and 0.02 M hydrochloric acid for 48 h.	The digested solution was mixed with 10x PBS, diluted with DW, and then pH was adjusted to 7.4 using 0.5 N sodium hydroxide.	Method: Self-assembly. Details:Incubating at 37 °C for 30 min. Concentration: 10 mg/mL.
Jakus, A. E. (2017, USA) [52]	Source: Porcine. Pre-Processing:Cut into pieces, rinsed multiple times in water, lyophilized, and cryomilled.	Technique:Immersion/Agitation. Detergents: 1 L of 0.5 % (w/v) SDS.	Enzyme/Acid:Dissolved in dichloromethane (4 mL per 0.3 g PLGA).	dECM was dissolved in dichloromethane, dried overnight, fabricated, rinsed in ethanol and sterile water, and dried overnight again.	Method: Self-assembly. Additives: PLGA. Concentration: 8 mL.
Jin, Y. (2018, South Korea) [53]	Source: Porcine. Pre-Processing:Cut into pieces, agitated in distilled water (24h), lyophilized and stored at 4 °C.	Technique:Immersion/Agitation. Detergents: 1 % (v/v) Triton X-100 and 0.1 % (v/v) ammonium hydroxide in distilled water (48h), followed by washing.	Enzyme/Acid:10 % (w/w) pepsin in 0.2 M HCl, stirring for 48 h at room temperature.	The lyophilized LEM was solubilized, washed with PBS, mixed with PBS and DI water, neutralized, and incubated at 37 °C for 30 min.	Method: Self-assembly. Additives: LEM hydrogel. Concentration: 10 mg/mL.
Jin, Y. Y. (2023, South Korea) [54]	Source: Porcine. Pre-Processing:Liver minced, frozen at −80 °C, washed in DI water with intervals, agitated at 150 rpm.	Technique:Immersion/Agitation. Detergents: 1 % Triton X-100, 0.1 % NH_3_·H_2_O, 1 % penicillin/streptomycin, and 0.01 mM PMSF for 3 days.	Enzyme/Acid:10 % pepsin (dECM:pepsin weight ratio 10:1) in 0.1 M HCl, agitated at RT for 48h.	Decellularized, digested by pepsin and HCl, then lyophilized.	Method: Chemical. Details: Agitated at RT for 48 h. Additives: Cu NZs@PLGA nanofibers.
Kellaway, S. C. (2023, United Kingdom) [55]	Source: Porcine. Pre-Processing:Liver stored at −20 °C, segmented into 3 mm slices, washed in dH_2_O three times for 15 min.	Technique:Immersion/Agitation. Detergents: 0.05 % EDTA at 37 °C, 3 % Triton X-100, 4 % sodium deoxycholate (1h each). Enzymes:0.02 % trypsin for 1h.	Enzyme/Acid:1 mg/mL pepsin in 0.01 N HCl.	B-ECM, LIV-ECM, SIS-ECM were lyophilized into powder, stored at 4 °C, then solubilized and neutralized.	Method: Not specified. Additives:Collagen I hydrogel. Concentration: 1g.
Khati, V (2022, Sweden) [20,56]	Source: Porcine. Pre-Processing:Frozen liver thawed to 4 °C, cut into 1 mm pieces, washed with DI water.	Technique:Immersion/Agitation. Detergents: SDS at increasing concentrations from 0.1 % to 1 %; Triton X-100 for 30 min.	Enzyme/Acid:Pepsin (10 % of dry tissue weight) in 0.5 M acetic acid for 48–72h to form a 3 % dLM solution.	Decellularized tissue washed, lyophilized, solubilized, then mixed with gelatin and crosslinker.	Method:Enzymatic crosslinking. Details: Mushroom tyrosinase and a polyethylene glycol-based crosslinker (xPEGx).
Khati, V (2022, Sweden) [56]	Source: Porcine. Pre-Processing:Frozen liver thawed to 4 °C, cut into 1 mm pieces, washed with DI water.	Technique:Immersion/Agitation. Detergents: SDS (0.1 %–1 %); Triton X-100 for 30 min.	Enzyme/Acid:Pepsin and acetic acid.	Decellularized liver was lyophilized to develop a 3–3.2 % dLM solution, adjusted to pH 7–7.4. xPEGx and mushroom tyrosinase were added for crosslinking.	Method:Enzymatic crosslinking. Details: Mushroom tyrosinase and a polyethylene glycol-based crosslinker (xPEGx).
Kim, M. (2023, South Korea) [57]	Source: Porcine. Pre-Processing:Chopped and washed with distilled water.	Technique:Immersion/Agitation. Detergents: 1 % v/v Triton X-100, 0.1 % v/v ammonia solution. Enzymes: 1.0 mg/mL proteinase K.	Enzyme/Acid:100 mg pepsin in 0.01 N HCl for 48h at 18 °C.	dECM powder was solubilized with HCl and pepsin, then PBS was added and neutralized with 0.5 N NaOH solution to prepare a dECM pre-gel.	Method: Physical crosslinking. Details: 40 mM CaCl_2_ selectively crosslinks alginate in 5 min; incubated at 37 °C for 25 min. Additives:Collagen hydrogel.
Kim, M.K. (2023, South Korea) [58]	Source:Decellularized human and rat liver tissue. Pre-Processing: Not specified.	Technique: Not specified.	Not specified.	dECM bioink was enhanced with gelatin to improve mechanical properties and used for 3D printing of liver lobule structures.	Method: Thermal and physical crosslinking. Details: Fully crosslinked dECM bioinks were used for testing. Additives: Gelatin-based bioink.
Kojima, H. (2023, Japan) [59]	Source: Porcine. Pre-Processing:Livers stored at −80 °C, thawed for 2 days at 4 °C, then rinsed with PBS.	Technique: Perfusion (Portal vein). Detergents:0.5 % SDS for 6h, 0.5 % Triton X-100, 0.05 % EGTA.	Enzyme/Acid:25 mg pepsin in 25 mL 0.01 M HCl for 72h at room temperature.	Decellularized pieces were lyophilized for 3 days, milled to powder, sterilized with 3 kGy of γ-rays, digested, neutralized with NaOH, and stored at 4 °C.	Method: Not specified (likely thermal). Details:Gelation observed only at ≥4 mg/mL. Concentration: 8 mg/mL.
Lee, J. (2014, Korea) [21]	Source: Rat, Mice, Human. Pre-Processing:Catheter inserted to portal vein, perfusion of cold distilled water for 30 min.	Technique: Perfusion (Portal vein). Detergents:1 % (v/v) Triton-X 100, 0.1 % (v/v) ammonium hydroxide for 4h.	Enzyme/Acid:10 % (w/w) pepsin in 0.1 M HCl for 48h at room temperature.	Lyophilized LEM was solubilized. Solution was mixed with 10x PBS, pH was adjusted with NaOH, and incubated for 30 min at 37 °C.	Method: Self-assembly. Details:Cross-linked by incubating at 37 °C for 40 min. Additives:Collagen I hydrogel. Concentration: 10 or 20 mg/mL.
Lewis, P. (2018, USA) [60]	Source: Porcine. Pre-Processing:Livers frozen at −80 °C, sliced into ∼1 mm cubes, washed in DI water.	Technique: Not specified. Detergents: 0.1 % SDS.	Enzyme/Acid:1 mg/mL pepsin in 0.01 M HCl for 48h.	After lyophilization, sterilized powder was digested at a concentration of 10 mg/mL dECM.	Method: Not specified, likely thermal. Concentration: 10 mg/mL.
Lewis, P. (2019, USA) [61]	Source: Porcine. Pre-Processing:Minced into ∼3 mm cubes, alternating washes of 1 mg/mL SDS and ultrapure water.	Technique:Immersion/Agitation. Detergents: 1 mg/mL SDS.	Enzyme/Acid:1 mg/mL pepsin in 0.01 M HCl for 48h.	dECM pieces were lyophilized, milled into a powder, digested in pepsin and HCl, and stored at −80 °C.	Method: Not specified, likely thermal. Concentration: 10 mg/mL.
Li, S. (2021, China) [16]	Source: Rat. Pre-Processing:Washed with PBS.	Technique: Perfusion (Route NR). Detergents:Perfused with 1 % SDS for 6h, rinsed with 1 % Triton X-100 for 30 min.	Enzyme/Acid:10 % (w/w) pepsin in 0.1 M HCl.	Decellularized tissue was lyophilized, milled into a powder, solubilized, and adjusted to 8 mg/mL.	Method: Not specified, gelation done at 37 °C. Concentration: 8 mg/mL. Additives:Hepatic non-acellular matrix (HNM).
Loneker, A. E. (2016, USA) [19]	Source: Rat, Human, Porcine, Canine. Pre-Processing: Sliced into ∼0.5 cm^2^ cubes.	Technique:Immersion/Agitation. Detergents: Triton X-100 (no more details). Enzymes: Trypsin/EGTA.	Enzyme/Acid:100 mg of pepsin in 100 mL of 0.01 M HCl for 24–72 h at room temperature.	ECM was lyophilized, cut, powdered, pepsin-solubilized, mixed with HCl, neutralized to pH 7.4, and incubated at 37 °C for ∼40 min.	Method: Not specified, likely thermal. Concentration: 8 mg/mL. Additives:UBM and SIS hydrogels.
Lu, S. (2018, China) [17]	Source: Rat. Pre-Processing: Frozen livers thawed, cannulated in portal vein, perfused with PBS for 1h at 20 mL/min.	Technique: Perfusion (Portal vein). Detergents:1 % (w/v) Triton X-100 for 30 min, then 0.1 % (w/v) SDS for 5h. Enzymes: 80 U/mL DNase and 5 U/mL RNase for 30 min.	Enzyme/Acid:0.5 mg pepsin in 0.1 M HCl for 72h at 250 rpm.	Lyophilized DLM powder was digested, reaction stopped with NaCl/10x PBS, pH adjusted to 7.0, and sterilized.	Method:Photocrosslinking. Details: 6.9 mW/cm^2^ UV light (365 nm) for 30s. Additives: GelMA hydrogel. Concentration: 1 mg.
M. Tabuchi (2015, Japan) [62]	Source: Rat. Pre-Processing: Sliced liver immersed in saline, packed in plastic bags, and pressurized to 980 MPa for 10 min.	Technique: High-hydrostatic pressure method. Detergents:None. Enzymes: DNase for 7 days, followed by 80 % ethanol.	Enzyme/Acid:100 mg pepsin in 45 mL of 0.1 N HCl for 48h at 37 °C.	Decellularized tissues were frozen, dried under reduced pressure, fractured using a food mill, and sifted into powder.	Not applicable (used as a powder mixed with fibrin glue).
Ma, X. (2018, USA) [14]	Source: Porcine. Pre-Processing:Cut into 0.5 cm^3^ pieces, cycles of 2h wash with hypotonic solution, rinsed three times with 1X D-PBS for 30 min each.	Technique:Immersion/Agitation. Detergents: 1 % SDS for 48h.	Enzyme/Acid:1 mg/mL pepsin in 0.1 M HCl for 24h at room temperature.	DECM was lyophilized, cryomilled, solubilized, neutralized, frozen, lyophilized again, and cryomilled into a fine powder.	Method:Photocrosslinking. Additives: Gelatin methacrylate (GelMA) and Collagen I hydrogels.
Milton, L. (2024, Australia) [63]	Source: Porcine. Pre-Processing:Tissue cut into 3 cm sections, homogenized, placed in orbital shaker at 100 rpm for 10 days with daily detergent change.	Technique:Immersion/Agitation. Detergents: Compared 4 protocols: 2 % Triton X-100; 2 % Triton X-100 followed by 2 % Triton X-100 with 0.1 % NH_4_OH; 2 % Triton X-100 and 0.1 % NH_4_OH combined; and 2 % SDS.	Enzyme/Acid:0.5 mg/mL pepsin in 0.01 M HCl for 48h.	Samples washed, frozen, lyophilized for 5 days, solubilized, and functionalized with thiol groups for click chemistry. Controls prepared with PEG-SH and PEG-MAL.	Method: Chemical. Details: Thiol-ene click chemistry by mixing dECM-SH/PEG-SH with 4-arm PEG-MAL. Concentration: 10 mg/mL.
Nakamura, S. (2014, Japan) [64]	Source: Rat. Pre-Processing: Blood removed by perfusion of 1 L calcium and magnesium-free (CMF) PBS for 10 min.	Technique: Perfusion (Portal vein). Detergents:2 L of 4 % Triton X-100 in CMF-PBS for 6h. Enzymes: 0.2 mg/mL DNase and 0.2 mg/mL RNase for 12h.	Enzyme/Acid:Pepsin in various HCl concentrations (0.001 M–6 M) for 72h.	Lyophilized dECM powder was added to HCl solution with or without pepsin for 72h, then insoluble components were removed by centrifugation.	Not applicable (used as a 2D film).
Nishiguchi, A. (2021, Japan) [65]	Source: Porcine. Pre-Processing:Tissue dissected, washed with saline, homogenized, incubated in PBS with 0.1 % peracetic acid and 4 % ethanol, washed, freeze-dried, and milled.	Technique:Immersion/Agitation. Detergents: 1 % Triton X-100/0.5 % SDS for 24h at 25 °C. Enzymes: DNase.	Enzyme/Acid:1 mL of pepsin solution (1 mg/mL) in 0.01 M HCl for 48h.	Powders were treated with DNase and pepsin solution to obtain solubilized matrix, which was then neutralized and freeze-dried.	Method: Chemical crosslinking with a pH-driven genipin gelator (GeniPEG).
Prebeg, T. (2023, Croatia) [66]	Source: Porcine. Pre-Processing:Frozen liver at −20 °C, cut into small pieces, immersed in a 0.9 wt% NaCl solution and agitated with mechanical stirrer at 400 rpm.	Technique:Immersion/Agitation. Detergents: Compared SLS or SLES (1 % or 0.5 %) for 30 or 60 min.	Enzyme/Acid:Pepsin for 48h.	Samples digested with pepsin for 48 h at pH 2, lyophilized, then mixed (8 mg material and 1 mL PBS).	Method: Not specified, likely thermal. Concentration: 8 mg/mL.
Rajabi, S. (2020, Iran) [67]	Source: Human, Sheep. Pre-Processing:Multiple rinsing with PBS, minced into small pieces, rinsed 5 times with DI water, freeze-thawed three times.	Technique:Immersion/Agitation. Detergents: 0.5 % SDS for 6h, then 1 % Triton X-100 for 10 min.	Enzyme/Acid:1 mg/mL pepsin in 0.5 M acetic acid for 72h.	Frozen decellularized tissues were lyophilized, powders soaked in 70 % ethanol, immersed in DI water, then re-lyophilized, digested, neutralized, and warmed to 37 °C for 10 min.	Method: Not specified, likely thermal. Concentration: 8 mg/mL.
Ravichandran, A. (2021, Australia) [68]	Source: Porcine. Pre-Processing:Liver chopped into 1 cm^3^ pieces, washed in DI water for one week at 4 °C with constant stirring, stored at −80 °C.	Technique:Immersion/Agitation. Detergents: Protocol 1: 0.5 % SDS for 3 days, then 1 % Triton X-100 for 2 days. Protocol 2: 1 % Triton X-100 + 0.1 % NH_4_OH for 5 days.	Enzyme/Acid:100 mg pepsin in 100 mL of 0.01 M HCl, stirred for 48h.	dECM was homogenized, lyophilized, solubilized, pH increased to 8 with NaOH, functionalized using methacrylation, and dialyzed against milliQ water.	Method:Photocrosslinking. Details: Thermal gelation was also performed. Concentration: 1g initial dECM.
Saheli, M. (2018, Iran) [13]	Source: Sheep. Pre-Processing:Frozen liver cut into 2 mm thick pieces, subjected to 5 mechanical agitations with DI water.	Technique:Immersion/Agitation. Detergents: 0.5 % SDS for 72h, then 1 % Triton X-100 solution for 30 min each time.	Enzyme/Acid:0.5 % pepsin in 0.5 M acetic acid (50 mg/mL), stirred with magnetic stirrer at 4 °C for 72h.	Decellularized tissue lyophilized, pepsin-digested, sterilized with UV, and pH neutralized with NaOH.	Method: Not specified, likely thermal. Additives:Collagen I hydrogel. Concentration: 3 mg/mL.
Sarika, N. (2020, Belgium) [69]	Source: Human. Pre-Processing:Liver fragments rinsed, cut into 0.5 cm^3^ pieces, frozen at −80 °C, thawed, and washed 3 times in DI water.	Technique:Immersion/Agitation. Detergents: 3 % Triton X-100 for 24h, 0.005 % EDTA. Enzymes: 0.02 % trypsin for 4h, 0.5 mg/mL DNase I.	Enzyme/Acid:1 mg/mL of pepsin in 0.01 M HCl, stirred for 48h at RT.	HL-ECM was solubilized with pepsin and HCl, stirred, neutralized, and salt concentration was balanced.	Method: Not specified, likely thermal. Concentration: 8 mg/mL.
Sasikumar, S (2022, India/Australia) [70]	Source: Others (Caprine). Pre-Processing: Chopped into ∼1 mm thick pieces.	Technique: Immersion/Agitation. Detergents: 0.5 % SDS for 48h, then 1 % Triton X-100 for 12h. Enzymes: DNase (5 U/mL) and RNase (5 U/mL) for 12h.	Enzyme/Acid: Pepsin (10 % of dECM powder weight) in 0.5 M acetic acid for 48h	Lyophilized dECM powdered with liquid nitrogen, digested, centrifuged, and neutralized to pH 7 with 10 M NaOH below 10 °C.	Method: Self-assembly. Details: Thermal gelation at 37 °C.
Serna-Márquez, N. (2020, Mexico) [71]	Source: Rat. Pre-Processing: Liver perfused with prechilled DI water at 5 mL/min for 30 min.	Technique: Perfusion (Portal vein). Detergents:1 % Triton-X 100, 0.1 % NH_4_OH. Enzymes: 10 μg/mL bovine pancreatic DNase.	Enzyme/Acid:1 mg of bovine gastric pepsin in 0.1 N HCl.	Lyophilized dECM pulverized with liquid N_2_, digested with pepsin and HCl, neutralized with NaOH, centrifuged, and stored at −70 °C.	Method: Not specified. Additives:Polyacrylamide hydrogels. Concentration: 10 mg.
Skardal, A. (2012, USA) [72]	Source: Porcine. Pre-Processing:Frozen liver pieces cut into 3 mm sections, shaken in 500 mL DI water on a rotary shaker at 200 rpm for 3 days at 4 °C.	Technique: Perfusion (Portal vein). Detergents:2 % Triton X-100 for 4 days, then 2 % TX-100 + 0.1 % NH_4_OH for 24 h.	Enzyme/Acid:100 mg Pepsin in 100 μL of 0.1 N HCl, incubated for 48h.	Fresh and decellularized tissues were lyophilized, mixed with pepsin, gamma irradiated, solubilized with HCl, and centrifuged.	Method: Self-assembly. Additives:Hyaluronic acid (HA) or heparin-conjugated HA, Collagen I. Concentration: 1g.
Skardal, A. (2015, USA) [82]	Source: Not specified. Pre-Processing:Tissues cut into 10 cm x 0.5–1.0 cm strips, minced, and shaken in distilled water for 3 days.	Technique:Immersion/Agitation. Detergents: 2 % Triton X-100 for 4 days, followed by 2 % TX-100 in distilled water with 0.1 % NH_4_OH for 24 h.	Enzyme/Acid:100 mg Pepsin in 100 mL of 0.1 N HCl.	Decellularized tissue was frozen, lyophilized, freezer milled to a powder, dissolved using HCl/pepsin, centrifuged, and filtered.	Method:Photocrosslinking. Details: Extralinker also used. Concentration: 1 g.
Tabatabaei Rezaei, N (2024, Canada) [73]	Source: Porcine liver. Pre-Processing: Not specified.	Technique: Not specified. Detergents: SDS-based decellularization followed by lyophilization.	Not specified.	Liver dECM underwent methacrylation followed by mixing with GelMA and photocrosslinking using visible light.	Method: Visible light photocrosslinking (DLP system). Additives: GelMA hybrid hydrogel. Concentration:0.5 %, 1 %, and 2 %.
Tabatabaei Rezaei, N (2025, Canada) [25]	Source: Porcine liver. Pre-Processing: Cut into <1 mm^3^ pieces, washed in distilled water (3 h), blended, centrifuged, lyophilized.	Technique:Immersion/Agitation. Detergents: 1 % (w/v) Triton X-100 for 24h, then 0.1 % (w/v) SDS for 48h.	Enzyme/Acid:Pepsin (10 mg/100 mg dECM) in 0.5 M acetic acid for 3 days.	Solubilized dECM was methacrylated, mixed with GelMA, and photocrosslinked.	Method: Visible light photocrosslinking (400–700 nm). Details: Eosin Y (0.02 mM) + triethanolamine (0.2 % w/v) photoinitiator. Additives: GelMA. Concentration:0.5–2 % LdMA in 5 % GelMA.
Takeda, Y. (2014, USA) [74]	Source: Others (Bovine liver). Pre-Processing: Cut into <1 cm^3^ pieces, thoroughly rinsed in DI water for 30 min.	Technique:Immersion/Agitation. Detergents: 1 % SDS for 3–5 days.	Enzyme/Acid:2 mg/mL pepsin in 0.1 M HCl for 2 days.	DECM rinsed overnight, mechanically broken into fibers, then either lyophilized or further digested into a gel-like material.	Method: Chemical. Details:Crosslinked via TG, EDC, or GA for 2D films; freeze-drying for 3D scaffolds.
W. Jeong (2021, South Korea) [75]	Source: Porcine. Pre-Processing:Liver chopped into 1–2 mm pieces and washed with distilled water.	Technique:Immersion/Agitation. Detergents: Compared SDS, SDC, TX, and TXA at 0.1 % and 1 % concentrations for 48h at 200 rpm.	Enzyme/Acid:100 mg pepsin per dECM powder weight in 0.1 N HCl.	DLM lyophilized, powder sterilized with 70 % ethanol, digested, and then neutralized.	Method: Thermal crosslinking. Details: Incubation at 37 °C for 30 min.
Willemse, J. (2022, Netherlands) [23]	Source: Human, Porcine. Pre-Processing:Thawed and flushed with 20–50 L of dH_2_O.	Technique: Perfusion (Portal vein & Hepatic artery). Detergents: 4 % Triton X-100 + 1 % NH_3_, followed by multiple cycles of 4 % Triton X-100. Enzymes: DNase solution.	Enzyme/Acid:10 % (w/w) Pepsin in 0.5 M Acetic Acid for 72h.	ECM was cut, freeze-dried for 72–96h, pulverized, digested, cooled on ice, neutralized, and diluted to 8 mg/mL.	Method: Self-assembly. Details:Thermal gelation. Concentration: 8 mg/mL.
Wolf, M. T. (2012, USA) [76]	Source: Porcine. Pre-Processing: Tissue cut into 1 cm^3^ pieces, washed in DI water.	Technique: Immersion/Agitation. Detergents: 0.1 % SDS for 24h, then 1 % Triton X-100 for 1h. Enzymes: DNase.	Enzyme/Acid: 1 mg/mL pepsin in 0.1 M HCl for 48h.	Lyophilized, milled to powder, solubilized, and neutralized.	Method: Self-assembly. Details: Thermal gelation. Concentration: 10 mg/mL.
X. Zhang (2015, China) [39]	Source: Rat. Pre-Processing:Freeze-thawed.	Technique: Perfusion (Portal vein). Detergents:0.5 % SDS for 12h, then 1 % Triton X-100 for 1h.	Enzyme/Acid:1 mg/mL pepsin in 0.1 M HCl.	Lyophilized to powder, solubilized, and neutralized.	Method: Self-assembly. Details:Thermal gelation.
Xinyi Li (2025, China) [77]	Not specified.	Not specified.	Not specified.	Not specified.	Not applicable (study on radiolabeled microspheres).
Xu, Z. (2025, China) [78]	Source: Mouse. Pre-Processing:Not specified.	Technique: Methanol-chloroform extraction. Enzymes: Trypsin digestion overnight at 37 °C.	Enzyme/Acid:Not for hydrogel formation; iTRAQ labeling and LC-MS/MS analysis for protein identification.	dECM was extracted, proteins identified via LC-MS/MS, modified with THBS1, and used as a bioink for organoid culture and transplantation.	Method: Not specified. Additives: Bioink containing THBS1-modified dECM.
Y. T. He (2020, China) [79]	Source: Porcine. Pre-Processing:Not specified.	Technique: Perfusion (Portal vein). Detergents:Triton X-100 (10 g/L) for 3h, then 10 g/L SDS, then 36 L of 10 g/L Triton X-100.	Enzyme/Acid:1 mg/mL porcine pepsin in 0.01 mol/L HCl for 48–72 h.	PLECM was cut into small pieces, lyophilized for 48 h, milled to powder, digested, and neutralized.	Method: Self-assembly. Details:Thermal gelation.
You, P. Y. (2024, China) [80]	Source: Porcine. Pre-Processing:Chopped into pieces of approximately 5 mm length/width and 2 mm thickness.	Technique:Immersion/Agitation. Detergents: 0.1 % v/v ammonia solution and 1 % v/v Triton X-100 at 200 rpm for 48 h.	Enzyme/Acid:LECM and pepsin in a ratio of 10 mg:1 mg in 0.5 mol/L HCl for 12h.	Lyophilized to make powder, then solubilized and neutralized.	Method: Physical crosslinking. Details: Ionic cross-linking with CaCl_2_. Additives:2 % Gelatin and 2 % Alginate. Concentration: Up to 100 mg/mL.
Yu, C. (2019, USA) [18]	Source: Porcine. Pre-Processing:Three rounds of freeze-thaw cycles in hypotonic solution, then rinsed with PBS.	Technique:Immersion/Agitation. Detergents: 1 % (w/v) SDS solution for 48 h.	Enzyme/Acid:1 mg/mL pepsin in 0.1 M HCl for 24 h at room temperature.	First mincing dECM finely, cryomilled into fine powder, digested, neutralized, lyophilized, then cryomilled again.	Method:Photocrosslinking. Details: LAP as photoinitiator. Additives: 5 % (w/v) GelMA. Concentration: 10 mg/mL (5 % w/v LdECM).
Zhang, J. (2025, China) [81]	Source: Porcine liver. Pre-Processing: Not specified.	Technique: Perfusion. Detergents: Not specified.	Enzyme/Acid:Acidic pepsin digestion (details not provided).	No details provided.	Method: Self-assembly. Details:Occurs at 37 °C. Concentration: 10 mg/mL.
Zou, C. Y. (2025, China) [24]	Source: Porcine small intestinal submucosa (SIS). Pre-Processing:Mechanical removal of tunica serosa/muscularis; lyophilization.	Technique: Multi-step process (mechanical excision + chemical detergents/enzymes).	Enzyme/Acid: Enzymatic digestion.	No details provided.	Method: Covalent (Schiff base, borate ester) + Non-covalent (H-bonding). Additives: Sodium alginate (ADA-PBA component).

#### 3.2.1 Source of tissue and decellularization techniques

The papers mentioned indicate that porcine liver has been the prominent material utilized in the creation of decellularized liver extracellular matrix (dLECM) that was utilized in approximately 51 studies. Such extensive use is a testimony to the accessibility and biological relevance [56,61,80]. Other tissues utilized included rat livers (n = 13), human specimens (n = 6), murine tissues (n = 2), as well as sheep tissues (n = 2). In certain instances, non-hepatic tissues such as porcine small intestine submucosa (SIS) and tissues from goats and cows were utilized as well [24]. Most tissues utilized came from healthy donators and were kept at −80 °C before further preparation.

Two approaches have been the overwhelming majority in the process of decellularization: immersion/agitation and perfusion. Immersion was cited the most often (53 articles) and was given preference due to the capability to process large quantities of diced or chopped tissues [38,63]. Perfusion approaches (19 articles) were utilized almost entirely on whole organs in efforts to preserve their vascular networks, often through the portal vein as a means for perfusion [21,31].

Before chemical detergents were introduced into service, the following physical pre-treatments were generally employed in the hope of making them more efficient. They comprised mechanical slicing or chopping (n = 22), freeze-thaw treatments (n = 13), homogenization (n = 6), and wash treatments that utilized PBS or deionized water (n = 9). Most frequently employed detergents were Triton X-100 (n = 48) and SDS (n = 36). These techniques were seldom employed alone; rather, investigators commonly used them in combination in tiered manners in order to optimize cell disruption. The most frequent combination was that of some form of initial chopping or cutting with some other technique. A classic example is that the majority of procedures combined slicing with freeze-thawing in order to both maximize surface area and break cells with ice crystal action (n = 66). Others combined slicing with lengthy hypotonic washing with the intention to osmotically lyse the cells (n = 17). This technique of stacking various physical pre-treatments emphasizes the empirical, "brute-force" technique that is meant to successfully compromise the integrity of the tissues in order to better permit penetration by the resultant chemical detergents.

Typically, these detergents were treated along with other chemicals such as sodium deoxycholate or ammonium hydroxide (NH_4_OH) that lysed the cellular and nuclear structures without perturbing the integrity of the extracellular matrix. Information on detailed concentrations as well as time intervals in the treatments is given in Table 2.

#### 3.2.2 Extracellular matrix solubilization and hydrogel formation

Subsequent to the decellularization process, tissues were conventionally subjected to freeze-drying, milling, and enzymatic processing to produce a soluble extracellular matrix (ECM) pre-gel solution. The predominant technique utilized for solubilization was acidic digestion employing pepsin. Hydrochloric acid (HCl) was employed as a buffering agent in 39 studies, while acetic acid was utilized in 17 instances. The concentrations of pepsin typically varied between 1 and 10 mg/mL, with the digestion period extending from 24 to 72 h [25,59]. After enzymatic digestion, the acidic environment was neutralized to reach a physiological pH range of 7.2–7.6, utilizing either NaOH or phosphate-buffered saline (PBS). Subsequently, incubation at 37 °C initiated thermal gelation, which was the most prevalent method for the preparation of hydrogels.

#### 3.2.3 Crosslinking formulations and strategies

To increase structural robustness and operability, researchers investigated alternative crosslinking methods for dLECM hydrogels. Photocrosslinking strategies were observed in nine submissions, commonly via the methacrylation of dECM in preparation for LdMA, followed by co-mixing with GelMA and polymerization via UV or visible light illumination. Innovative work by Tabatabaei Rezaei et al. [21,22] was the creation of visible-light photocrosslinking, permitting rapid, cell-compatible gel formation in 1 min—potentially highly beneficial in high-magnification 3D bioprinting. Other methods included chemical crosslinking (e.g., glutaraldehyde; [14,25,73]), enzymatic crosslinking (e.g., mushroom tyrosinase; [20]), and physical methods such as ionic interactions [38] or freeze-thaw cycling [22].More innovation on hybrid systems is the production of proteolytically stable hydrogels by mixing soluble liver ECM with self-assembling, synthetic dipeptides. Biswas et al. demonstrated that a hydrogel composed of isoleucine-α,β-dehydrophenylalanine (IAF) and soluble liver ECM (sLEM) formed a biocompatible as well as stable 3D microenvironment that could accommodate the long-term culture of the primary hepatocytes, surpassing one prominent limitation characteristic to purely biological matrices [33]. Moreover, other groups fabricated composite hydrogels by mixing dLECM with polymers such as alginate, chitosan, or PVA, enabling the increase of printability as well as the preservation of the mechanical strength. An inventory of these methods is provided in Table 2.

### 3.3 Applications and biological outcomes

The practical use of dLECM hydrogels has been depicted in various scenarios, including cell culture platforms as well as *in vivo* regeneration studies. Table 3 presents a comprehensive description on the experimental setups, the methods applied, and the biological outcomes.

**Table 3:** Summary of study designs, applications, and biological outcomes.

Author (Year)	Study Design & Application	Cell Types Used	Key Functional Assays & Outcomes	Primary Outcome & Main Results	*In Vivo* Model Details (Animal, Disease, Follow-up)
Agarwal, T (2018) [26]	*In vitro*; Cell culture, Organoid culture.	HepG2 cells.	Biochemical:Higher albumin and GAG synthesis on CLECM-coated surfaces. Cytocompatibility (MTT): Showed minimal cell death (<5 %) after 24h.	Primary Outcome:To evaluate CLECM as an efficient biomaterial platform for tissue engineering. Main Results:CLECM-coated surfaces promoted a highly differentiated and polarized phenotype, enhanced hepatocyte marker expression, and supported microvasculature formation.	Not Applicable.
Agarwal, T (2019) [27]	*In vitro*; Cell culture, Organoid culture.	HepG2 cells, HUVECs.	Cytocompatibility (MTT): Consistent increase in viability and proliferation. Immunogenicity:Scaffolds did not elicit any significant immunogenic response.	Primary Outcome:To demonstrate the superiority of CLECM-S over conventional Col-S scaffolds. Main Results: The scaffolds increased functional hepatocyte markers in HepG2 cells and demonstrated pro-angiogenic properties.	Not Applicable.
Ahmed, E (2020) [28]	*In vivo* & *in vitro*; Cell/Organoid culture, *In vivo*implantation.	Not specified.	Biochemical: TAA-induced elevation of ALT and AST levels were significantly lowered by LECM-AgNP treatment. Albumin expression was higher. Histology: Ki67-positive cells were significantly increased in treated groups.	Primary Outcome:To evaluate the therapeutic effects of an LECM hydrogel-AgNP mixture on TAA-induced liver injury. Main Results:The mixture improved histological liver regeneration, reduced IL-6 and TGF-β, and highly expressed albumin and hepatocyte growth factor.	Animal: Rat. Disease Model:Thioacetamide (TAA)-induced acute liver failure (white necrotic foci). Follow-up: Not specified.
Almalla, A (2023) [29]	*In vitro*; Cell culture, Organoid culture.	HepaRG cells.	Cytocompatibility (Live/Dead):Showed similar adhesion, proliferation, and viability compared to cultures on pure rat tail collagen gels.	Primary Outcome:To establish papain as a cost-effective and efficient alternative to pepsin for dECM solubilization. Main Results: Papain-digested dECM preserved gelation properties, showed enhanced bioadhesiveness, and supported HepaRG viability and proliferation similar to collagen.	Not Applicable.
Almalla, A (2024) [30]	*In vitro*; 3D bioprinting, Cell/Organoid culture.	Not specified.	Cytocompatibility (Live/Dead): A higher proportion of dead cells was observed in bioresins with increasing LAP photoinitiator concentration.	Primary Outcome:To assess the printability and cytocompatibility of dECM-GMA for advanced biofabrication. Main Results: dECM-GMA showed faster photocrosslinking, lower final stiffness, and faster enzymatic biodegradation. Cell proliferation was enhanced in softer dECM-GMA constructs.	Not Applicable.
B. Wang (2018) [15]	*In vitro*; Cell culture.	Bone marrow-derived MSCs (BM-MSCs).	Cytocompatibility (Live/Dead): Most cells were alive during culture. Differentiation:Viability and hepatogenic differentiation were significantly enhanced compared to non-coated plates.	Primary Outcome:To investigate the ability of dLECM hydrogel to facilitate BM-MSC proliferation and hepatic differentiation. Main Results: The liver ECM coating performed as well as or better than Matrigel, inducing additional metabolic functions.	Not Applicable.
B. Wang (2020) [31]	*In vivo* & *in vitro*; In vivo implantation.	Not applicable.	Histology: ECM-only and EG sponges showed good biocompatibility with smooth liver surface, reduced wound size, and accelerated regeneration, whereas gelatin-only sponges showed severe inflammation.	Primary Outcome:To create and assess composite ECM/gelatin (EG) sponges for *in vivo* applications. Main Results: Increasing gelatin reduced porosity and swelling but enhanced degradation resistance. Increasing ECM improved porosity and swelling.	Animal: Rat. Disease Model:Liver tissue wound (3 sharp incisions). Follow-up: 7 and 14 days.
Beachley, V (2018) [32]	*In vivo* & *in vitro*; Not specified.	Not specified.	Not specified.	Primary Outcome:To develop an alternative formulation for digestion of tissue ECM-based hydrogels. Main Results: ECM particle-GAG composite hydrogels are injectable and avoid digestion procedures that alter ECM properties.	Not Applicable.
Bhatt, S (2025) [22]	*In vitro*; Tissue engineering, 3D bioprinting.	HepG2 cells.	Biochemical:Albumin synthesis and urea metabolism increased over 5 days, highest in crosslinked scaffolds. Cytocompatibility:High cell viability and proliferation on PVA/CH + dLM-FT scaffolds.	Primary Outcome:To develop and characterize a decellularized caprine liver matrix (dLM)-based hydrogel for liver tissue engineering. Main Results: Successfully fabricated biocompatible hydrogels with enhanced mechanical properties suitable for 3D bioprinting and supporting hepatocyte function.	Not Applicable.
Biswas, S (2022)(33)	*In vitro*; 3D cell culture for liver tissue engineering.	Huh7 cells, primary rat hepatocytes (pHCs).	Cytocompatibility (MTT): High viability. Function: pHCs on hybrid gel showed significantly higher albumin (11.5x) and urea (3.1x) secretion vs. collagen control on day 7. Morphology: Confocal Z-stacking confirmed 3D cell migration into the gel.	Primary Outcome: To develop a proteolytically stable hybrid scaffold for long-term culture of pHCs. Main Results: The dipeptide-sLEM hybrid hydrogel provides a stable, biomimetic 3D microenvironment that preserves pHC viability and enhances hepatocyte-specific functions for extended periods.	Not Applicable.
Bobrova, M. M. (2021) [34]	*In vivo* & *in vitro*; Cell/Organoid culture, *in vivo*implantation.	Mouse fibroblasts.	Cytocompatibility:Significant increase in the number of mouse fibroblasts in LDLF samples compared to control.	Primary Outcome:To assess the regenerative potential of decellularized rat liver film (LDLF). Main Results: LDLF had a high regenerative potential, attributed to its sinuous, rough, and highly nanoporous structure.	Animal: Rat. Disease Model:Skin wound. Follow-up: 3, 18, 23, 40 days.
Bual, R. P. (2019) [35]	*In vitro*; 3D bioprinting, Cell/Organoid culture.	Primary rat hepatocytes.	Function: L-ECM II hydrogel supported higher liver-specific functions and wider cell spread.	Primary Outcome:To establish intact L-ECM as an effective substrate for liver tissue engineering. Main Results: L-ECM II (a specific preparation) had higher ECM protein content, denser fiber network, and higher compression strength.	Not Applicable.
Chen, J. (2023) [4]	*In vitro*; Cell/Organoid culture, Drug screening.	Primary cholangiocytes.	Cytocompatibility (Live/Dead):Cholangiocytes in decellularized liver scaffolds (DLS) formed a continuous bile duct network with high viability, which was not observed in dECM-HG culture.	Primary Outcome:Generation of functional ductal organoids (FDOs) for disease modeling and drug screening. Main Results: The cholangiocytes in FDOs demonstrated high viability and expressed specific biomarkers.	Not Applicable.
Coronado, R. E. (2017) [36]	*In vitro*; Cell culture.	Porcine hepatocytes.	Cytocompatibility (MTT): Used to assess hepatocyte viability on the decellularized matrices.	Primary Outcome:Evaluation of two decellularization methods (Triton X-100 vs. NaDOC). Main Results: Both methods were effective, but Method B (NaDOC) showed better preservation of ECM architecture and collagen content.	Not Applicable.
Coronado, R. E. (2019) [37]	*In vitro*; Cell/Organoid culture, Drug screening.	Hepatocyte-like cells (HLCs) derived from stem cells.	Gene Expression:HLCs on pLECM showed higher upregulation of genes for drug transport and metabolism compared to collagen I, but lower than primary hepatocytes.	Primary Outcome:To assess decellularized liver substrate for differentiating stem cells into HLCs. Main Results: HLCs cultured on pLECM-AA displayed typical hepatocyte morphology, but could not fully replace primary hepatocyte functionality.	Not Applicable.
D. S. Sun (2018) [38]	*In vitro*; Cell culture.	HCCLM3 cells (Hepatocellular carcinoma cell line).	Viability:Enhanced cell viability in DLM-ALG beads compared to ALG beads alone. Function: MMP and plasminogen activator (PA) activity were higher in cells cultured in DLM-ALG.	Primary Outcome:To investigate the viability, proliferation, and metastatic potential of HCCLM3 cells in DLM-Alginate beads. Main Results: DLM-Alginate beads enhanced cell viability and cancer-related enzyme activity.	Not Applicable.
D. Y. Zhang (2015) [39]	*In vitro*; Cell culture.	Muscle precursor cells (MPCs).	Differentiation:The number of differentiated myotubes was significantly increased on muscle-ECM substrates compared to other gels.	Primary Outcome:To develop a culture system for expansion and myo-differentiation of MPCs on various tissue ECMs. Main Results: MPC proliferation was significantly enhanced on ECM-HA-HP substrates, with the greatest effect from muscle ECM.	Not Applicable.
D. Udagawa (2024)(11)	*In vivo* & *in vitro*; Implantation, Cell culture.	Primary human hepatocytes (PXB cells).	Biochemical:Human albumin increased linearly over time *in vivo*. Histology: Showed enhanced cell-cell interactions and angiogenesis in L-ECM gel vs. collagen gel.	Primary Outcome:To investigate orthotopic transplantation of mature hepatocytes using a dECM hydrogel in a fibrotic liver model. Main Results: L-ECM gel successfully engrafted transplanted hepatocytes and supported hepatic function.	Animal: Rat. Disease Model:Thioacetamide (TAA)-induced liver fibrosis. Follow-up:1 and 2 weeks.
Damania, A. (2017) [14]	*In vivo* & *in vitro*; Liver tissue engineering.	HepG2 cells, primary human hepatocytes.	Biochemical: A 20–60 % improvement in liver function parameters was observed post-implantation. Histology:Scaffolds showed complete integration with the native tissue.	Primary Outcome:To use dECM-coated cryogel scaffolds for bioartificial liver support systems and implantable constructs. Main Results: The scaffolds maintained hepatocyte functionality *in vitro*and demonstrated efficacy in improving liver failure conditions *in vivo*.	Not specified.
Deegan, D. B. (2015) [40]	*In vitro* & *in vivo*; Cell/Organoid culture, *in vivo*implantation.	Primary human hepatocytes.	Cytoskeletal Organization:Increased gel stiffness correlated with increased cell attachment and cytoskeletal organization.	Primary Outcome:To investigate how gel stiffness affects primary human hepatocyte function. Main Results: Cell attachment, viability, and cytoskeletal organization improved with increased stiffness, but long-term phenotype maintenance was uncertain.	Not Applicable.
Di Gravina, G. M. (2023) [41]	*In vitro*; 3D bioprinting, Cell/Organoid culture, Drug screening.	Not specified.	Cytocompatibility:High cell viability was observed post-printing.	Primary Outcome:To develop a lyo-dECM powder as a biomimetic component for 3D-printable hybrid hydrogels. Main Results: Adding dECM powder did not alter the viscoelastic properties of the original material, and the composite was successfully bioprinted with cells.	Not Applicable.
Dziki, J. L. (2017) [42]	*In vitro*; Cell culture.	Macrophages.	Macrophage Phenotype:Macrophages exposed to liver ECM did not significantly change M1/M2 marker expression, unlike ECM from other tissues (e.g., SIS, dermis). Viability:Macrophage viability was assessed.	Primary Outcome:To assess the effects of ECM from eight different source tissues on macrophage phenotype and function. Main Results: ECM source differentially influences macrophage polarization (pro-inflammatory vs. pro-remodeling).	Not Applicable.
Elomaa, L. (2020) [43]	*In vitro*; 3D bioprinting, Cell/Organoid culture.	Not specified.	Not specified.	Primary Outcome:To replace GelMA with native organ-derived materials in hybrid resins for 3D printing. Main Results: The presence of PCL-MA in the hybrid resins improved the 3D printing fidelity compared to neat GelMA resins.	Not Applicable.
G. S. van Tienderen (2023) [44]	*In vitro*; Organoid culture, Drug screening.	Cholangiocyte and cholangiocarcinoma organoids.	Not specified.	Primary Outcome:To create size-standardized organoids for scalable production using a hybrid microcapsule system. Main Results: The established system can reduce size variability in organoid culture by providing a uniform scaffolding.	Not Applicable.
Gao, Y. (2023) [45]	*In vitro*; Liver tissue engineering.	Hepatocytes.	Biochemical:Albumin production and CYP enzyme activity were significantly enhanced on aligned dECM-coated scaffolds. Morphology:Improved hepatocyte adhesion and morphology.	Primary Outcome:To develop electrospun scaffolds combining nanotopography and dECM to enhance hepatocyte functionality. Main Results: The combination of aligned nanofibers and dECM coating significantly improved hepatocyte function.	Not Applicable.
Guagliano, G. (2022) [46]	*In vitro*; 3D bioprinting, Cell/Organoid culture.	Not specified.	Cytocompatibility (Live/Dead, MTT): The presence of ECM in "Hep3Gel" favored cell adhesion and growth, increasing cell viability by almost 400 %.	Primary Outcome:To assess the applicability of "Hep3Gel" as a tool for developing 3D *in vitro* models of the liver. Main Results:The addition of ECM doubled the survival rate of cells embedded within control hydrogels after 4 days.	Not Applicable.
H. Ijima (2018) [47]	*In vitro*; Other (physical property quantification).	Not applicable.	Not applicable.	Primary Outcome:To quantify the physical properties of L-ECM and L-ECM gels. Main Results:L-ECM yielded lower elasticity than collagen I. An increase in gel concentration resulted in a decrease in biodegradation rate and an increase in mechanical strength.	Not Applicable.
Hou, Y. T. (2020) [48]	*In vivo* & *in vitro*; Cell/Organoid culture, *in vivo*implantation.	Hepatocytes.	Biochemical:Albumin and urea production in Glt-HPA-DLM groups were superior to negative controls. Cytotoxicity: The hydrogel rescued hepatocytes from toxicity induced by GaIN, CHCl_3_, and CCl_4_.	Primary Outcome:To assess decellularized liver matrix as a substrate for rescue of acute hepatocyte toxicity and fibrosis. Main Results: The Glt-HPA-DLM hydrogel was effective for hepatocyte culture and showed promising effects against hepatic fibrosis *in vivo*.	Animal: Rat. Disease Model:Hepatic fibrosis and liver cirrhosis. Follow-up: 1, 3, 5 days/weeks (not clear).
Hussein, K. H. (2020) [49]	*In vivo* & *in vitro*; 3D bioprinting, Cell/Organoid culture, *in vivo*implantation.	Hepatocytes, LX-2 cells.	Biochemical:Albumin levels were restored to near-normal after 3 weeks. Cytocompatibility (MTT): Liver hydrogel showed higher proliferation than negative control.	Primary Outcome:To evaluate liver hydrogel as an injectable biomaterial for liver tissue engineering. Main Results: The hydrogel improved TGF-β1-induced LX-2 cell activation, reduced fibrosis, and promoted recovery to a nearly normal structure *in vivo*.	Animal: Mice. Disease Model:Liver fibrosis. Follow-up: 7 and 21 days.
Ijima, H. (2019) [50]	*In vitro* & *in vivo*; Cell culture, Organoid culture, Transplantation.	Hepatocytes.	Biochemical: High albumin synthesis activity *in vitro*. Viability: 51 % viability at day 21 *in vitro*, 18 % survival rate *in vivo*.	Primary Outcome:To assess the effectiveness of L-ECM gel as a substrate for hepatocyte culture and transplantation. Main Results:Hepatocytes in L-ECM gel formed a large liver tissue-like structure *in vivo*, twice the size of those in collagen gel.	Not specified.
Jakus, A. E. (2017) [52]	*In vitro*; 3D bioprinting, Cell/Organoid culture.	Not specified.	Cytocompatibility (Live/Dead):Imaging confirmed cell viability.	Primary Outcome:To create "Tissue Papers" from organ-specific dECM for future use. Main Results: All tissue paper types supported cell adhesion, viability, and proliferation over four weeks.	Not Applicable.
Jin, Y. (2018) [53]	*In vitro*; 3D bioprinting, Cell/Organoid culture, Drug screening.	Not specified.	Cytocompatibility (MTT, Live/Dead):Assessed. Function:Improved hepatic functionalities, metabolic activity, biosynthetic activity, and drug responses.	Primary Outcome:To use a combined strategy of direct reprogramming, matrix engineering, and microfluidics. Main Results: The platform improved hepatic functionalities and drug responses.	Not Applicable.
Jin, Y. Y. (2023) [54]	*In vivo* & *in vitro*; ALF therapy.	Hepatocyte-like cells (HLCs).	Cytocompatibility (Live/Dead): HLCs showed good cell viability. Histology:Mice treated with HLCs/Cu NZs@fiber/dECM showed less tissue necrosis and more cell proliferation.	Primary Outcome:To use Cu NZs@PLGA nanofiber-reinforced dECM for HLCs as a promising approach for ALF therapy. Main Results: The composite hydrogel protected transplanted HLCs, improved their hepatic functions, and showed excellent synergistic therapeutic efficacy in ALF mice.	Animal: Mice. Disease Model:Acute Liver Failure (ALF).
Kellaway, S. C. (2023) [55]	*In vivo* & *in vitro*; Peripheral nerve regeneration.	DRG neurons, Schwann cells.	Histology: Nerve tissue with dense axons had infiltrated the conduits and grafts *in vivo*.	Primary Outcome:To use decellularized tissue hydrogels for the regeneration of peripheral nerves. Main Results: B-ECM and SIS-ECM hydrogels induced cellular alignment and showed axonal regeneration comparable to collagen controls.	Animal: Rat. Disease Model:Sciatic nerve transection. Follow-up: 28 days.
Khati, V (2022) [56]	*In vivo*; 3D bioprinting.	HepG2 cells.	Biochemical:Albumin secretion increased 12-fold by day 7. AFP and KRT19 expression also increased. Viability: Dropped to 85.8 % by day 7.	Primary Outcome:To develop a dLM hydrogel suitable for bioprinting under physiological conditions. Main Results: The crosslinked dLM showed a 16-fold increase in viscosity and a 32-fold increase in storage modulus.	Not Applicable.
Khati, V (2022) [20]	*In vitro*; 3D bioprinting.	HepG2 cells, NIH 3T3 cells.	Biochemical:Albumin and urea secretion were 4-fold higher by day 7, with co-culture showing better results than monoculture. Viability: Dropped to ∼90 % by day 7.	Primary Outcome:To use dLM hydrogel in indirect 3D bioprinting of a robust hepatic construct. Main Results: Fibroblast co-culture improved viability, albumin secretion, and liver-specific gene activity.	Not Applicable.
Kim, M. (2023) [57]	*In vitro*; 3D Bioprinting.	Primary mouse hepatocytes (PMH).	Biochemical:Albumin (4.3x), Urea (2.5x), and CYP1A2 activity (2x) were higher in dECM group. Drug Response: 1.8-fold increased responsiveness to hepatotoxic drugs.	Primary Outcome:To enhance cell-cell and cell-ECM interactions using dECM-incorporated hepatocyte spheroids. Main Results: The spheroids maintained shape and viability and showed significant improvements in hepatic function and gene expression.	Not Applicable.
Kim, M.K. (2023) [58]	*In vitro*; 3D bioprinting.	Hepatocytes, HUVECs.	Biochemical:Albumin synthesis, urea production, and CYP enzyme activity confirmed. Toxicity:Acetaminophen toxicity test performed. Viability: High viability of both cell types in bioink.	Primary Outcome:To develop a mechanically enhanced dECM bioink (dECM-gBioink) for bioprinting functional liver lobule-like structures. Main Results: Blending with gelatin improved mechanical properties, enabling successful printing of liver structures with enhanced function.	Not Applicable.
Kojima, H. (2023) [59]	*In vivo* & *in vitro*; Cell therapy.	Mesenchymal stem cells (MSCs).	Biochemical:Increased RNA expression of HGF and TSG-6, especially with TNFα stimulation. Viability (MTT):Remained high (99.5 %) after 4 days.	Primary Outcome:To enhance the efficacy of cell therapy in pancreatitis with a combinational treatment of dECM hydrogel and MSCs. Main Results: The combination therapy showed therapeutic effects on inflammation and fibrosis in a rat pancreatitis model.	Animal: Rat. Disease Model:Dibutyltin dichloride (DBTC)-induced pancreatitis. Follow-up: 2 weeks.
Lee, J. (2014) [21]	*In vivo* & *in vitro*; Cell culture, *in vivo* implantation.	Hepatocytes, hADSCs.	Biochemical:Albumin secretion and urea synthesis were improved in LEM vs collagen I. Viability (Live/Dead):Viability was higher in LEM hydrogel vs collagen I after 1 week.	Primary Outcome:To demonstrate the feasibility of dECM for both 2D coating and 3D hydrogel platforms in liver tissue engineering. Main Results: LEM hydrogel showed improved elastic properties, rapid gelation, enhanced hepatocyte function, and upregulated hepatic gene expression.	Animal: Athymic mice. Disease Model: Not applicable (ectopic implantation). Follow-up: 7 days.
Lewis, P. (2018) [60]	*In vitro*; Cell culture, Organoid culture.	Cholangiocytes.	IHC: Staining confirmed maturation of tight junctions (E-cadherin + ZO1).	Primary Outcome:To form a complex bile duct network within dECM hydrogels. Main Results: The hydrogels supported tight junction formation and maturation, and clonal branching of cholangiocytes into multi-colored structures.	Not Applicable.
Lewis, P. (2019) [61]	*In vitro*; 3D bioprinting, Cell culture.	Biliary epithelium, hepatocyte spheroids.	Imaging: Showed formation of 3D biliary trees and alignment of ducts based on strut width.	Primary Outcome:To direct the growth and alignment of biliary epithelium within dECM hydrogels using 3D bioprinting. Main Results: Successfully fabricated a true multi-layer dECM structure, and showed that seeding sequence impacts resulting structures.	Not Applicable.
Li, S. (2021) [16]	*In vivo* & *in vitro*; Cell culture, *in vivo* implantation.	BRL-3A cells, macrophages.	Biochemical: AST & ALT levels decreased in IRI rats treated with HAM hydrogels. Albumin and urea concentration increased. Viability: >95 % viability for BRL-3A cells.	Primary Outcome:To attenuate hepatic damage caused by ischemia/reperfusion injury (IRI) using hepatic acellular matrix (HAM). Main Results: HAM hydrogel regulated macrophage polarization from M1 to M2 via TLR4/NF-κB signaling, exerting a hepatoprotective effect.	Animal: Rat. Disease Model:Ischemia/reperfusion injury in liver. Follow-up: 7 days.
Loneker, A. E. (2016) [19]	*In vitro*; Cell culture.	Primary rat hepatocytes (PRHs).	Biochemical:Increased bile and albumin production. Morphology:Formation of multinucleate cells.	Primary Outcome:To determine if solubilized liver ECM could better maintain hepatocyte phenotype compared to collagen I or non-liver ECM. Main Results: The highest maintenance of phenotype was observed in hepatocytes supplemented with canine and porcine LECM.	Not Applicable.
Lu, S. (2018) [17]	*In vitro*; Liver-on-a-chip model.	HepG2 cells.	Viability: Increased in hybrid DLM-GelMA hydrogels for up to 7 days. Biochemical:Enhanced hepatocyte function.	Primary Outcome:To develop a biomimetic liver tumor-on-a-chip model using dECM. Main Results: The integration of DLM components with GelMA increased cell viability and enhanced hepatocyte function compared to GelMA only.	Not Applicable.
M. Tabuchi (2015) [62]	*In vivo* & *in vitro*; Myocardial infarction therapy.	Not specified.	Histology: dLIV powder + fibrin glue preserved residual myocardium and induced greater neovascularization compared to controls. Viability (WST-8): Assessed.	Primary Outcome:To examine the use of decellularized tissue powder in a rat model of acute myocardial infarction. Main Results:Decellularized liver powder promoted cell integration and neovascularization *in vivo*, suppressing myocardial necrosis.	Animal: Rat. Disease Model:Acute Myocardial Infarction (MI). Follow-up: 7 days.
Ma, X. (2018) [14]	*In vitro*; 3D bioprinting, Cancer modeling.	HepG2 cells.	Viability (Live/Dead):Similar viability in liver dECM and collagen I scaffolds.	Primary Outcome:To develop a photocrosslinkable dECM platform for studying Hepatocellular Carcinoma (HCC) progression. Main Results: When encapsulated in dECM scaffolds with cirrhotic stiffness, HepG2 cells showed reduced growth and high invasion markers, enabling modeling of stromal invasion.	Not Applicable.
Milton, L. (2024) [63]	*In vitro*; Cell culture.	HepG2, HepaRG cells.	Biochemical:Enhanced metabolic activity, CYP1A2/CYP3A4 function, and excretory capacity (MRP2 staining, FDA excretion assay) vs. monolayer and collagen. Viability:Cytocompatible, except for SDS-decellularized gels.	Primary Outcome:To develop "click-functionalized" dECM hydrogels as a highly controlled alternative to conventional tissue hydrogels. Main Results: The click-dECM hydrogels were cytocompatible and supported enhanced hepatic function.	Not Applicable.
Nakamura, S. (2014) [64]	*In vitro*; Cell culture.	Hepatocytes.	Biochemical:Albumin production of hepatocytes was higher on ECM film compared to collagen I film.	Primary Outcome:To investigate how HCl concentration affects the molecular structure and function of solubilized ECM. Main Results: Liver ECM composition is an effective hepatocyte culture substratum, superior to collagen I for albumin production.	Not Applicable.
Nishiguchi, A. (2021) [65]	*In vitro*; Tissue adhesive development.	Not applicable.	Not specified.	Primary Outcome:To develop a dECM-based tissue adhesive using a genipin gelator. Main Results: dECM hydrogels crosslinked with GeniPEG formed within seconds and exhibited greater tissue adhesive strength than those crosslinked with genipin.	Not Applicable.
Prebeg, T. (2023) [66]	*In vitro*; Decellularization protocol comparison.	Not applicable.	Not specified.	Primary Outcome:To compare sodium lauryl sulfate (SLS) and sodium lauryl ether sulfate (SLES) for liver decellularization. Main Results: The natural architecture of the tissue was partially preserved depending on the detergent, with SLES causing less damage.	Not Applicable.
Rajabi, S. (2020) [67]	*In vitro*; Cardiac microtissue fabrication.	HUVECs, Cardiac Progenitor Cells (CPCs).	Viability: Maximal cell viability for HUVECs in all hydrogels. Gene Expression (qRT-PCR):Cardiomyogenic genes significantly upregulated in CPCs cultured on cardiac matrix (D-CM).	Primary Outcome:To develop cardiac microtissues using dECMs from different tissues. Main Results:Tissue-specific dECM directed cell differentiation, with cardiac ECM promoting cardiomyogenesis and aortic ECM promoting endothelial gene expression.	Not Applicable.
Ravichandran, A. (2021) [68]	*In vitro*; 3D liver models.	Not specified.	Viability (Live/Dead):Protocol 2 (Triton + NH_4_OH) supported viable cell spheroids for 1 week; Protocol 1 (SDS) led to cellular death.	Primary Outcome:To develop more controlled 3D *in vitro*liver models. Main Results: Different decellularization protocols yield hydrogels with distinct mechanical properties and cytocompatibility.	Not Applicable.
Saheli, M. (2018) [13]	*In vitro*; Organoid culture.	Hepatocarcinoma cells.	Gene Expression:Significant upregulation of ALB, CYP3A4, etc. Function: More ALB and AAT secretion, urea production, and CYP3A4 activity in 3D LEM gel vs. controls. Viability:Majority of cells were viable after 10 days.	Primary Outcome:To demonstrate that a 3D liver-derived hydrogel promotes the function of liver organoids. Main Results: LEM hydrogel organoids showed significantly enhanced liver-specific functions compared to 2D culture or collagen gels.	Not Applicable.
Sarika, N. (2020) [69]	*In vitro*; Cell culture.	Human liver cells.	Proteomics:Analysis revealed proteomic diversity, type 1 collagen abundance, and partial loss of integrity after solubilization.	Primary Outcome:To improve ECM to provide cells with an *in vitro* environment that resembles native tissue. Main Results:The impact of solubilized HL-ECM on the phenotype or functionality of human liver cells was limited.	Not Applicable.
Sasikumar, S (2022)(70)	*In vitro*; Development of a model for drug-induced liver injury (DILI) screening	HepG2 cells, HUVECs, LX2s (in coculture).	Drug Toxicity (MTT): Assessed response to acetaminophen (APAP), levofloxacin, and trovafloxacin. Function: DLM enhanced expression of CPS1 (urea cycle) and BSEP (polarity marker).	Primary Outcome: To evaluate the influence of liver ECM in predicting DILI. Main Results: A multi-cellular coculture system within the DLM hydrogel showed the highest sensitivity to hepatotoxins and was able to correctly identify the toxicity of trovafloxacin, a drug that fails in standard preclinical models.	
Serna-Márquez, N. (2020) [71]	*In vitro*; Cell culture.	Primary hepatocytes.	Function:Hepatocytes had poor adhesion when collagen I was absent. Collagen I inhibited apoptosis and activated ERK. Albumin expression was not preserved.	Primary Outcome:To investigate the role of fibrillar collagen I in hepatocyte survival and aggregation on soft hydrogels. Main Results: Fibrillar collagen I plays a key role in hepatocyte survival on soft hydrogels.	Not Applicable.
Skardal, A. (2012) [72]	*In vitro*; Cell culture.	Hepatocytes.	Function:Hepatocytes in HA/HP groups containing LTE or LEE synthesized steady levels of albumin and urea and sustained CYP450 metabolism. Viability (Calcein-AM): Highest in heparin-containing groups.	Primary Outcome:To enhance hepatocyte survival and function by combining dECM extracts with hyaluronic acid (HA) or heparin (HP). Main Results:Combining LEE with heparin-conjugated HA significantly increased hepatocyte metabolism and function.	Not Applicable.
Skardal, A. (2015) [82]	*In vitro*; 3D bioprinting.	Not specified.	Biochemical: ECM solutions were analyzed for growth factor content.	Primary Outcome:To develop a hydrogel bioink toolkit for mimicking native tissue properties. Main Results: The toolkit allowed for mimicking of native tissue biochemical and mechanical properties.	Not Applicable.
Tabatabaei Rezaei, N (2024) [73]	*In vitro*; 3D bioprinting, liver microenvironment models.	Hepatocytes.	Biochemical:Albumin production confirmed. Viability: High cell viability observed over 28 days.	Primary Outcome:To develop a photocrosslinkable liver dECM (LdECMMA) bioink for high-resolution 3D bioprinting. Main Results: The bioink supported hepatocyte proliferation and functionality and showed improved printability using visible light.	Not Applicable.
Tabatabaei Rezaei, N (2025) [25]	*In vitro*; 3D bioprinting, liver tissue modeling.	HepG2 cells.	Biochemical:Albumin secretion increased by 40 % vs. GelMA. CYP1A2 was upregulated. Drug Response:Improved drug response (APAP/CYP1A2).	Primary Outcome:To develop and characterize a photocrosslinkable liver dECM-GelMA hybrid hydrogel. Main Results: The LdMA-GelMA hydrogel showed tunable mechanics and enhanced HepG2 proliferation, spheroid formation, and liver-specific functions.	Not Applicable.
Takeda, Y. (2014) [74]	*In vitro*; Cell culture.	HepG2 cells.	Viability (MTT):The number of cells in the scaffolds was analyzed.	Primary Outcome:To create 2D films and 3D scaffolds with controlled properties using reconstituted dECM. Main Results: Not detailed.	Not Applicable.
W. Jeong (2021) [75]	*In vitro*; 3D bioprinting.	Not specified.	Biochemical: SDS and SDC detergents severely damaged GAG and elastin proteins.	Primary Outcome:To investigate the effects of different decellularizing detergents on dECM bio-inks. Main Results: TXA (Triton X-100 + ammonium hydroxide) based dECM bio-ink possessed the highest ECM content among all bio-inks tested.	Not Applicable.
Willemse, J. (2022) [23]	*In vitro*; Organoid culture.	Cholangiocyte organoids.	Differentiation:Differentiation into hepatocyte-like cells remained intact, although proliferation rates were lower than in traditional BME hydrogels.	Primary Outcome:To investigate if dLECM hydrogels can replace mouse tumor-derived BME for organoid culture. Main Results: Liver ECM-derived hydrogels support the growth and differentiation of human cholangiocyte organoids.	Not Applicable.
Wolf, M. T. (2012)(76)	*In vitro*; Cell culture.	Primary rat hepatocytes.	Function: Albumin and urea production were maintained for 7 days. Viability: High viability observed.	Primary Outcome: To evaluate dLECM hydrogel for primary hepatocyte culture. Main Results: The hydrogel supported hepatocyte viability and function, outperforming collagen I.	Not Applicable.
X. Zhang (2015) [39]	*In vitro*; Cell culture.	Adipose-derived stem cells (ADSCs).	Not specified.	Primary Outcome:To compare liver dECM as a coating matrix for hepatogenic differentiation of ADSCs with other ECM components. Main Results:Solubilized liver matrix provided a better biomimetic environment and enhanced hepatogenic differentiation of ADSCs than collagen, fibronectin, or Matrigel.	Not Applicable.
Xinyi Li (2025) [77]	*In vivo*; HCC treatment.	Not applicable.	Cytotoxicity: ICT and R848 showed dose-dependent cytotoxicity. Immune response:Increased CD8^+^ T cell infiltration in tumor and spleen.	Primary Outcome:To develop and evaluate^131^I-labeled PLGA microspheres for HCC treatment. Main Results: The radiolabeled microspheres achieved targeted delivery, induced tumor cell death, activated an immune response, and inhibited tumor growth.	Animal: Rat. Disease Model:Hepatocellular carcinoma (HCC). Follow-up: 15 days.
Xu, Z. (2025)(78)	*In vivo* & *in vitro*; Organoid reprogramming, Liver regeneration.	Hepatocyte organoids.	Biochemical:Albumin and urea synthesis were elevated in THBS1-treated groups. Histology: Grafts showed proliferation (Ki-67) and differentiation (SOX9).	Primary Outcome:To enhance hepatocyte organoid function and liver regeneration via thrombospondin-1 (THBS1)-modulated dECM. Main Results: THBS1-modulated matrix enhanced organoid integration and improved mouse liver regeneration post-transplantation.	Animal: Mouse. Disease Model:Liver injury (post-transplantation). Follow-up: Not specified.
Y. T. He (2020) [79]	*In vitro*; Cell culture, Organoid culture.	Primary hepatocytes, MSCs.	Gene Expression:High expression of ALB, CYP450 markers, and urea cycle genes. Function:Demonstrated long-term survival and stable functionality via sustained ALB and urea production.	Primary Outcome:To create rat hepatocyte organoids as an *in vitro* model for drug testing. Main Results:Primary hepatocytes and MSCs self-assembled into 3D hepatocyte organoids with stable, long-term functionality.	Not Applicable.
You, P. Y. (2024) [80]	*In vivo* & *in vitro*; Bone tissue engineering, 3D bioprinting.	Rat bone marrow stem cells (rBMSCs).	Viability (Live/Dead):Assessed after hydrogel preparation. Histology: Staining confirmed angiogenesis and osteogenesis.	Primary Outcome:To investigate an rBMSC-laden LdECM–gelatin–alginate scaffold in a rat model of critical-sized calvarial defects. Main Results: The scaffolds facilitated angiogenesis and osteogenesis at the defect site.	Animal: Rat. Disease Model:Critical-sized calvarial defects. Follow-up: 4 weeks.
Yu, C. (2019) [18]	*In vitro*; 3D bioprinting.	Not specified.	Not specified.	Primary Outcome:To use dLECM as a bioink for scanningless and continuous 3D bioprinting. Main Results: Not detailed.	Not Applicable.
Zhang, J. (2025) [81]	*In vivo*; Acute liver failure treatment.	MSC spheroids, HUVECs.	Biochemical:Lowest AST levels in the SpEV-shielded group. Albumin upregulated in HLCs. Histology:Improved tissue repair, minimal fibrosis, and neovascularization.	Primary Outcome:To evaluate the efficacy of spheroid-derived extracellular vesicle (SpEV)-shielded artificial liver lobules (ALL) in treating acute liver failure. Main Results: The construct enhanced angiogenesis, reduced necrosis, inflammation, and improved liver regeneration.	Animal: BALB/c mice. Disease Model: CCl_4_-induced acute liver failure (ALF). Follow-up: 3 days.
Zou, C. Y. (2025) [24]	*In vivo* & *in vitro*; Hemostasis, Tissue repair.	Not specified.	Biochemical:Normal AST/ALT, ALB/TP levels restored at 28 days. Hemostasis:Reduced blood loss by 76 % vs. control. Histology:Promoted angiogenesis and bile duct formation.	Primary Outcome:To develop an all-in-one ECM powder for hemostasis and in-situ tissue functional repair. Main Results:The powder self-assembled in 5s into a robust adhesive hydrogel, effectively controlled severe hemorrhage, and promoted functional liver regeneration.	Animal: New Zealand white rabbits. Disease Model: Liver volume defect (hemorrhage). Follow-up: 28 days.

#### 3.3.1 Biological performance and cytocompatibility in vitro

Out of the total 74 studies, 35 focused on *in vitro* usage. Overwhelming majority opinions among the scientists asserted that dLECM hydrogels provide a better hepatic microenvironment compared with traditional 2D cultures or one-component gels like collagen I.

Decellularized liver ECM hydrogels were commonly used as substrates for *in vitro* cell culture (n = 3235). In these experiments, hydrogels invariably supported high cell compatibility, with viability above 90 % as determined by Live/Dead, MTT, and CCK-8 assays [16,26]. High cytocompatibility was a near-standard finding. Of the studies that carried out quantitative viability assays, virtually all declared cell viability levels above 90 %. However, the decision on the detergent utilized in the process of decellularization was found critical. For example, Ravichandran et al. (2021) and Milton et al. (2024) represented a key counterpoint, reporting that hydrogels from harsh SDS-based methods resulted in extensive cell death. A major endpoint measured in 28 studies was the retention of hepatocyte-specific functions. An overwhelming majority—26 of these studies (93 %)—claimed that dLECM hydrogels increased dramatically functions such as albumin secretion, urea synthesis, and cytochrome P450 activity compared to controls. For example, Biswas et al. (2022) documented that primary hepatocytes on their hybrid gel secreted 11.5 times more albumin compared with the collagen control 1, and Wolf et al. (2012) demonstrated sustained function that improved over collagen I. In a significant exception, Sarika et al. (2020) discovered their human-derived dLECM had just a 'limited' effect, emphatically highlighting that favorable outcomes are not always the rule and are very dependent on processing protocols [13,21,25,63].

#### 3.3.2 Applications in 3D bioprinting and organoid culture

Owing to the characteristic rheological as well as bioactive properties, the dLECM hydrogels have become popular as bioinks for 3D bioprinting. Fourteen articles cited their usage in printing complicated, cell-based constructs, the shear thinning and fast gelation allowing high resolution printing [18,58].

Nineteen studies have explored their use in organoid culture, wherein the biomimetic microenvironment supported the spontaneous self-organization of stem cells and hepatocytes into complex structures, including bile duct networks and hepatocyte spheroids [61,79]. Importantly, Xu et al. (2025)(78) determined that hepatocyte organoid reprogramming was greatly increased by the introduction of THBS1-modified dECM bioink, and engraftment was enhanced following transplantation. In addition to regeneration, dLECM hydrogels are becoming center stage as the principal vehicles for the development of more predictive *in vitro* models of drug-induced liver injury (DILI). A paradigmatic article by Sasikumar et al. demonstrated that a hepatocyte-containing multicellular coculture comprising endothelial and stellate cells in a caprine-derived dLECM hydrogel could reliably discern the hepatotoxicity of trovafloxacin—a drug that had passed regular preclinical testing before causing disastrous liver failure in human patients. This characterizes the importance of including a tissue-specific microenvironment with the interaction between cells as a path forward toward the development of credible toxicological models [70].

#### 3.3.3 In vivo regenerative outcomes

Out of the 26 studies that conducted *in vivo* experiments, the outcomes can be classified into three general positive results. First, nearly all the studies demonstrated exemplary biocompatibility, such that scaffolds demonstrated effective integration with host tissues and limited fibrotic encapsulation compared with controls between days 7–14 [28].

Second, the majority of the studies that stained for vascular markers showed vigorous angiogenesis, as indicated by prominent CD31 staining. Third, in hepatic injury models, several studies showed functional repair, as shown by the fact that dLECM hydrogels suppressed inflammatory reactions, delayed fibrosis progress, and aided the recovery of the markers of liver function such as AST and ALT [16]. An especially interesting work by Zou et al. (2025) proposed a dECM-based powder that, by itself, self-assembled into a hydrogel adhesive on contact, effectively stopped bleeding in the model of hemorrhage in the rabbit liver and fostered the repair of tissues [24]. More information is given in Table 3.

## 4 Discussion

The results from this systematic review, based on the evidence from 74 studies, highlight the revolutionary capability of dLECM-based hydrogels in liver tissue engineering. Such biomaterials succeed in accurately mimicking the native liver microenvironment's biochemical and structural complexity and provide a very bioactive surface for hepatocyte culture, 3D bioprinting, organoid formation, and *in vivo* regeneration [22,81]. Data indicate a very innovative field characterized by fast innovation and methodological variety, especially regarding decellularization methods, dECM solubilizability, and hydrogel formulation. However, the heterogeneity, although testifying to a very wide exploration of the best conditions, is also a very serious impediment to standardization and clinical translation.

One key observation from this review is the extreme methodological heterogeneity in dLECM construction, which directly hinders interspecies comparisons across studies and hinders the creation of a gold-standard protocol. Detergent selection, for example, constitutes a stubborn trade-off between the efficiency of decellularization and preservation of the ECM(75). Harsh ionic detergents such as SDS are very effective at clearing away cellular and nuclear matter but are globally documented as disrupting the architecture of the ECM and reducing vital components such as GAGs and growth factors [75,76]. On the other hand, gentler non-ionic detergents such as Triton X-100 better maintain the bioactivity of the ECM but frequently produce incomplete decellularization, such that combination with other agents or enzymes is needed [37,79]. This trade-off, however, is never absolute and is seen, in particular, as being strongly dependent on the subtleties of the whole protocol. For example, Sasikumar et al. (2022) utilized sequential treatment with 0.5 % SDS followed by 1 % Triton X-100 and documented respectable retention levels of both collagen (∼60 %) and GAGs (∼61 %), such that the hydrogel was functionally superior for DILI modeling [70]. This result indicates that variables such as the detergent concentration, time of exposure, sequentiality, and the comprehensiveness of the washing steps are all-important variables that, taken as a group, define the eventual final bio-functionality of the matrix. Underreporting of these minute details across studies frequently constitutes a significant impediment to reproducibility and renders determination of clear attribution of biological outcomes to specific protocol decisions challenging.

The solubilization process of dECM is another site of high methodological convergence and divergence. Most studies depend on pepsin digestion under acidic conditions (usually 0.01–0.5 M HCl or acetic acid) for 24–72 h, a process that efficiently disrupts collagen networks into injectable precursors [21,56]. Yet, the time and enzyme-to-tissue ratio significantly deviate, the former potentially affecting the extent of protein cleavage and preservation of bioactivity [64]. For example, Tabatabaei Rezaei et al. (2025) employed pepsin at 10 mg per 100 mg dECM for 72 h [25], whereas others employed the same under increased concentrations or times [59]. This heterogeneity is likely the reason why there have been conflicting accounts regarding growth factor preservation, such as that of hepatocyte growth factor (HGF) and fibroblast growth factor (FGF), both critical in the function and regeneration of hepatocytes [82]. Finally, the common method of lyophilating dECM into the powdered form as illustrated by Di Gravina et al. (2023), Khati et al. (2022), and Tabuchi et al. (2015) is a significant stepping stone toward off-the-shelf status and batch-to-batch uniformity, the latter allowing for persistent storage and reproducible reconstitution [41,56,62].

With respect to hydrogel formation, thermal self-assembly at 37 °C is still the most common, making use of natural fibrillogenesis of collagenous matrices [21]. This method is appealing given its simplicity and cytocompatibility, as illustrated in the work of Hussein et al. (2020), Bobrova et al. (2021), and Prebeg et al. (2023)(34, 49, 66). However, the mechanical properties of such physically crosslinked hydrogels are frequently inadequate for load-bearing or implantation on the timescale of days or lifetime [76]. In response, some works have introduced chemical and enzymatic crosslinking methods. For instance, Zou et al. (2025) fabricated a dual-crosslinked hydrogel via Schiff base and borate ester linkages, and van Tienderen et al. (2023) utilized enzymatic crosslinking via horseradish peroxidase and H_2_O_2_, allowing high-speed gel formation with increased stability [24,44]. These methods provide enhanced mechanical integrity at the expense of cytotoxicity risks from residual crosslinkers, highlighting the importance of optimization with caution [14]. Despite the majority reporting positive results, the point is that the bioactivity of dLECM is far from certain and entirely dependent on the whole processing chain. An interesting work by Sarika et al. (2020) revealed that their solubilized human liver ECM exerted just a "limited" effect on the phenotype and function of primary human liver cells, a resounding contrast with the notable enhancements that the majority of other works have expressed [69]. In pointing up this contradiction, we highlight a number of possible pitfalls: the solubilization protocol itself, if too vigorous, will denature key bioactive proteins and leave the final hydrogel more like a structural scaffold than a true mimetic [64]. In addition, the origin of the issued tissue (e.g., human as opposed to porcine) as well as the donative status of the issued human will cause significant variability in the initial composition of the issued ECM [19]. The combination with other compounds can have unexpected results; e.g., Biswas et al. (2022) found that the addition of soluble liver extracellular matrix (ECM) into their synthetic dipeptide gel, despite enhancing biocompatibility, surprisingly reduced its mechanical strength from around 7.2 kPa down to about 2.8 kPa [33]. In aggregate, these findings highlight the-important caveat that decellularized liver ECM (dLECM) is not a one-size-fits-all biomaterial, but is instead a complex system whose performance is dependent on the meticulous optimization of each step, from procurement of the tissues through the final formulation as a hydrogel.

The functional results recorded in the studies considered invariably show the high bioactivity of dLECM hydrogels compared with artificial or one-component matrices [76]. Encapsulation of hepatocytes, HepG2 cells, or stem cell-derived hepatocyte-like cells in dLECM hydrogels results in the significant enhancement of albumin production, urea production, and hepatic gene expression, such as cytochrome P450 enzymes, alpha-fetoprotein (AFP), and cytokeratin 19 (CK19) [13,22]. Significantly, Lewis et al. (2018) demonstrated the formation of the complex bile duct networks inside dLECM hydrogels, highlighting the ability of the matrix to guide morphogenesis [60]. Similarly, Zhang et al. (2025) demonstrated that dLECM-based artificial liver lobules appreciably increased the function of the liver in acute liver failure models, leading to the reduction of AST levels and promoting neovascularization [81]. Such findings suggest that dLECM not only provides structural support but serves as an active reservoir for signaling, acting on the behavioral response of the cells and promoting the repair of the tissues [72].Even with these encouraging outcomes, numerous conflicting data and methodologic deficiencies need critical consideration. Foremost, residual DNA content—a major predictor of the risk of immunogenicity—ranges substantially across studies [19]. Whereas certain studies indicate values significantly below the 50 ng/mg threshold (e.g., Hou et al., 2020), others withhold data such that comprehensive safety evaluation is not possible [49]. This variability underscores the adequacy of the decellularization protocols and possible host immune rejection *in vivo* [42]. Also, the characterization is often neglected. Comparatively, few studies release data on rheologic properties, such as storage modulus (G′) or gel formation kinetics, such that hydrogel stiffness cannot be correlated with hepatocyte functionality, or specific designs can be developed on particular ends [40]. Lastly, and perhaps worst, is the over-reliance on tissues from animals, particularly porcine and rodents, such that the data have limited translational relevance [31]. Whereas Sarika et al. (2020) have successfully handled human dLECM-based constructs, such investigations remain the exception, and species-based differences in the constituent composition of the ECM could have a significant bearing on the response of the cells [19,69].

Another new and very promising trend is that towards the use of dLECM hydrogels in 3D bioprinting and organoid engineering. Exemplar studies by Khati et al. (2022), Tabatabaei Rezaei et al. (2025), and Kim et al. (2023) have shown that dLECM can be developed into bioinks with tunable rheometric properties, allowing the printing of structurally intricate, functionally active, and potentially implantable liver constructs [20,25,58]. In addition, dLECM has been shown to have the potential as a better-than-Matrigel alternative culture system for organoid cultures that provides a xenogeneic-free, tissue-specific niche that supports the growth of cholangiocyte and hepatocyte organoids without the risk profile seen with tumor-derived matrices [23]. Integration with synthetic polymers (e.g., gelatin, alginate, GelMA) or bioactive compounds (e.g., THBS1, AgNPs) further extends the repertoire, allowing the material to be used in antimicrobial therapy, immunomodulation, and controlled drug release [28,81]. Translating dLECM hydrogels from the bench to the bedside faces multiple tangible translational challenges beyond method standardization [8]. Key among these is the challenge of immunogenicity [14]. The significant variability in residual DNA content that is reported, ranging from below the 50 ng/mg safety limit through to levels substantially in excess, presents a direct risk of causing adverse host immune reactions [19,80]. In addition, for xenogeneic products, and especially porcine-derived xenogeneic products, the presence of non-human oligosaccharide epitopes such as the galactose-α-1,3-galactose (α-Gal) epitope is known to cause robust hyperacute rejection cascade [31]. Another key challenge is scalability [32]. Present academic-scale production, frequently dependent upon tedious, labor-based, hand-tissue-processing, is inadequate for the creation of the large, consistent, GMP-compliant, production quantities needed when performing clinical trials and commercial production [52]. Overcoming this will require automated, industrial-scale decellularization and processing units [9]. Encouraging directions forward may be hybrid systems that reduce these very significant challenges. As one example, the Biswas et al. (2022) method utilizes a simple, scalable, and proteolytically stable synthetic dipeptide as the chief structural unit of the hydrogel, requiring only a small percentage of bioactive soluble Dlecm [33]. Such methods, uniting the biological realism of dLECM with the reproducibility and scaleability that is typical in synthetic material, hold the potential as the brightest hope toward therapeutically useful and marketable liver tissue engineering treatments [6].

## 5 Conclusion

In conclusion, dLECM-based hydrogels is a versatile and robust platform in liver-engineering, able to maintain cell viability, promote hepatic function, and facilitate sophisticated uses like bioprinting and regenerative therapy. Yet the field needs to go beyond proof-of-concept towards standardization, scale-up, and strict *in vivo* validation. For the future, the creation of GMP-compatible protocols, the employment of human-derived dECM, and thorough determination of long-term safety and efficacy should be given high priorities. Only then can the true clinical potential of dLECM hydrogels be actualized in the treatment of liver disease and the promotion of regenerative medicine.

## Consent to participate

Not applicable.

## Ethical approval

Not applicable.

## Availability of data and material

Not applicable.

## Consent for publication

Not applicable.

## Author contributions

Study concept and design: ZM, MMZ; Data extraction: HF and HAR; Drafting of the manuscript; MMS and HF, and Critical revision of the manuscript: ZM, MMS, IS, and MMZ

## Funding

The authors did not receive any funding to conduct the present study.

## Declaration of competing interest

The authors declare that they have no known competing financial interests or personal relationships that could have appeared to influence the work reported in this paper.
